# Gut microbiota modulation of epigenetic target EHMT2: *Lacticaseibacillus rhamnosus* Fb7-311 regulated renal cell carcinoma apoptosis and metastasis

**DOI:** 10.1038/s12276-026-01659-6

**Published:** 2026-03-05

**Authors:** Jeongmin Lee, Jinkwon Lee, In Hwan Tae, Yunsang Kang, Jinsan Kim, Tae Young Ryu, Haneol Yang, Su-gi Lee, Kunhyang Park, Doo-Sang Park, Cho-Rok Jung, Jung Hwa Lim, Moo-Seung Lee, Dae-Soo Kim, Mi-Young Son, Hyun-Soo Cho

**Affiliations:** 1 Korea Research Institute of Bioscience and Biotechnology, Daejeon, Republic of Korea; 2 Korea University of Science and Technology, Daejeon, Republic of Korea; 3 School of Medicine, Sungkyunkwan University, Suwon, Republic of Korea

**Keywords:** Metastasis, Oncogenes, Cancer models

## Abstract

Renal cell carcinoma (RCC), also known as renal cell cancer or renal cell adenocarcinoma, is the most common type of kidney cancer and a common malignant tumor of the urinary system. Although surgical interventions and cancer treatments such as chemotherapy are commonly used to treat RCC, the persistently low 5-year survival rate and high relapse rate underscore the continued need to identify novel therapeutic targets for the development of new RCC treatments. Here, in this study, we therefore identified EHMT2 overexpression using RCC RNA-sequencing data derived from The Cancer Genome Atlas database. EHMT2 knockdown induced apoptosis and inhibited the migration/invasion of the A498 and Caki-1 RCC cell lines. Treatment with BIX-01294, an EHMT2-specific inhibitor, similarly suppressed growth and inhibited the migration/invasion of RCC cell lines, confirming the functional role of EHMT2 in RCC progression. Furthermore, DDIT3 was identified as a direct downstream target of EHMT2 and its expression was upregulated following EHMT2 downregulation by epigenetic regulation. Moreover, 3D spheroid models and in vivo experiments supported the therapeutic potential of EHMT2 as a therapeutic target for RCC treatment. In addition, by screening the components of the gut microbiota capable of modulating the EHMT2–DDIT3 axis, we identified *Lacticaseibacillus rhamnosus* Fb7-311, whose culture supernatant promoted apoptosis through regulation of the EHMT2–DDIT3 pathway. Thus, the development of EHMT2-specific inhibitors and the therapeutic application of *L. rhamnosus* Fb7-311 may represent promising strategies for effective treatment and clinical guidance of RCC treatment.

## EHMT2 inhibition and gut microbiota show promise in RCC

Kidney cancer is a major global health issue, with renal cell carcinoma (RCC) being the most common type. This study explores how gut bacteria might help treat RCC. Researchers focused on a protein called EHMT2, which is linked to cancer growth. They found that reducing EHMT2 expression in RCC cells increased cell death and decreased cell migration and invasion. EHMT2 expression was suppressed following treatment with the gut bacterium *Lacticaseibacillus rhamnosus*. Bacterial supernatant reduced cancer cell growth by modulating EHMT2 and an EHMT2 direct target, DDIT3, which promotes cell death. The study employed cultured cancer cells and animal models to evaluate these effects. Overall, the results suggest that targeting EHMT2 with bacteria or drugs could represent a novel therapeutic strategy for RCC. Future research may further explore gut bacteria as treatments for kidney cancer.

This summary was initially drafted using artificial intelligence, then revised and fact-checked by the author.

## Introduction

Kidney cancer represents a substantial global health burden, necessitating continued research efforts to improve our understanding of this malignancy^1^. Renal cell carcinoma (RCC) is the most common type of kidney cancer, accounting for approximately 90% of all kidney cancer cases^2^. The incidence of RCC has steadily increased over the past decades, with clear cell RCC representing the majority of all RCC cases^2^. Various treatments are available for RCC, including radiotherapy, immunotherapy and surgery^2^. However, despite these treatments, advanced RCC remains largely incurable, with a 5-year survival rate of less than 20% for patients with metastatic disease^3,4^, highlighting the urgent need for novel therapeutic approaches.

Epigenetic modifications, such as acetylation, methylation, phosphorylation and ubiquitination of histones, play crucial roles in cancer development and progression^5^. These modifications regulate chromatin structure and gene expression without altering the DNA sequence itself^6^. In RCC, aberrant histone methylation and acetylation patterns have been extensively documented and associated with tumor progression and a poor prognosis, as specifically reflected in the substantially shorter survival of patients with low levels of H3K4me2 and H3K18ac^7^. In addition, alterations in H3K9 methylation have been reported to modulate RCC pathogenesis^8^. Euchromatin histone lysine methyltransferase 2 (EHMT2), also known as G9a, catalyzes the mono- and dimethylation of histone H3 at lysine 9 (H3K9me1/2), which are marks associated with transcriptional repression^9^. Epigenetic modifications induced by EHMT2 are implicated in various cancer types, including gastric cancer, pancreatic cancer, hepatocellular carcinoma and RCC^10–12^. In cancer cells, EHMT2 promotes tumorigenesis through multiple mechanisms, including the silencing of tumor suppressor genes^13^, the promotion of epithelial‒mesenchymal transition (EMT) through its interaction with Snail^14^ and the maintenance of cancer stem cell properties^15^.

The gut microbiota, a diverse community of microorganisms inhabiting the human intestine, plays a critical role in host physiology, including the regulation of inflammation, immune responses, and the gut‒brain axis^16,17^. When dysbiosis or an imbalance in the gut microbiota occurs, it can contribute to various pathological conditions, such as obesity^18^, nonalcoholic fatty liver disease^19^, inflammatory bowel disease^20^ and cancer^10,21^. Recently, the modulation of the gut microbiota has emerged as a promising approach for the treatment of various types of cancer, including RCC^22^. The gut microbiota influences cancer development and progression through multiple mechanisms, including the production of metabolites and the shaping of inflammatory and immune responses^23^. Accumulating evidence suggests that specific bacterial strains can exert antitumor effects. Among them, *Lacticaseibacillus rhamnosus* has been shown to induce apoptosis in cancer cells^24,25^, enhance antitumor immunity through dendritic cell activation and T cell responses^26^ and produce cell-free supernatants with anticancer properties^27^. Furthermore, *L. rhamnosus* has been shown to enhance the antitumor efficacy of PD-1 immunotherapy by promoting the dendritic cell-mediated production of IFNα and IFNβ through the activation of the cGAS–STING pathway^28^, suggesting its potential as a novel therapeutic agent or adjuvant in cancer treatment.

The relationships among the gut microbiota, epigenetic modifications and cancer cell death pathways represent an emerging area of research with substantial therapeutic potential. Recent studies have provided insights into how microbial metabolites can influence the host epigenetic machinery^29^, suggesting that probiotics might exert anticancer effects through epigenetic mechanisms^30–32^. However, the specific mechanisms by which beneficial bacteria such as *L. rhamnosus* modulate epigenetic regulators in cancer cells remain largely unexplored.

Thus, in this study, we demonstrate that EHMT2 plays a critical role in RCC cell survival and that its inhibition leads to DDIT3-mediated apoptosis. We revealed that the knockdown of EHMT2 inhibits cell proliferation and induces apoptosis in RCC cell lines while also suppressing EMT. Similar anticancer effects are observed upon treatment with the EHMT2 inhibitor BIX-01294 (BIX). Our mechanistic studies reveal that EHMT2 epigenetically silences DDIT3 through H3K9me2 modification at its promoter region and that the inhibition of EHMT2 relieves this repression, leading to DDIT3-induced apoptosis. Importantly, we demonstrate that the *L. rhamnosus* Fb7-311 supernatant (Sup) downregulates EHMT2 expression and induces DDIT3-mediated apoptosis in RCC cells. These findings are further validated in both three-dimensional (3D) spheroid culture systems and a xenograft model, where EHMT2 inhibition notably suppressed tumor growth. This study revealed the EHMT2–DDIT3 axis as a novel therapeutic target in RCC and demonstrated that this pathway can be modulated by *L. rhamnosus*, suggesting potential applications for both pharmacological and microbiota-based therapeutic approaches.

## Materials and methods

### Cell culture and reagents

The human RCC cell lines A498 and Caki-1 were purchased from the Korean Cell Line Bank and cultured in RPMI-1640 supplemented with 10% fetal bovine serum (FBS) and 1% penicillin/streptomycin in a humidified atmosphere with 5% CO_2_ at 37 °C. BIX (ab141407) was purchased from Abcam.

### Cell viability assay

Cell Counting Kit-8 (CCK-8; Dojindo Laboratories) was used to assess cell viability. A498 cells were seeded in 6-well plates at 1.5 × 10^5^ cells/well, and Caki-1 cells were seeded in 6-well plates at 2 × 10^5^ cells/well and incubated for 24 h. After BIX treatment or siRNA transfection for 48 h, Fb7-311 *L. rhamnosus* treatment for 24 h or indole-3-carbinol treatment for 72 h, CCK-8 solution and RPMI-1640 medium supplemented with 10% FBS were added to each well and the cells were incubated at 37 °C with 5% CO_2_ for 5 min. The absorbance was measured at 450 nm using a microplate reader. For crystal violet (CV) staining, the cells were fixed with methanol for 5 min and stained with 0.1% CV after BIX treatment or siRNA transfection for 48 h, Fb7-311 *L. rhamnosus* treatment for 24 h or indole-3-carbinol treatment for 72 h.

### Bacterial culture

The *L. rhamnosus* Fb7-311 strain was obtained from the Bio R&D Product Program (https://biorp.kribb.re.kr/, BP1184523). The bacterial strain was cultivated in de Man, Rogosa and Sharpe (MRS) media (BD) under anaerobic conditions at 37 °C for 36 h. The bacterial culture was incubated at 65 °C for 30 min for pasteurization and centrifuged at 3,000*g* for 10 min. The supernatant was collected in a fresh new tube and stored at −70 °C until use.

### siRNA transfection

A498 and Caki-1 cells were seeded in plates and incubated for 48 h before siRNA transfection. The targeting or control siRNAs (Bioneer Co. Ltd.) were transfected into the cancer cell lines at a concentration of 100 nM using RNAiMax (Invitrogen) for 48 h. The sequences of the siRNAs used were as follows: siCont (5′-AUGAACGUGAAUUGCUCAATT-3′, 5′-UUGAGCAAUUCACGUUCAUTT-3′), siEHMT2 #1 (5′-GCAAAUAUUUCACCUGCCA-3′, 5′-UGGCAGGUGAAAUAUUUGC-3′), siEHMT2 #2 (5′-CCUCUUCGACUUAGACAACAA-3′, 5′-UUGUUGUCUAAGUCGAAGAGG-3′) and siDDIT3 (5′-CUGACUACCCUCUCACUAG-3′, 5′-CUAGUGAGAGGGUAGUCAG-3′).

### RT–qPCR

Total RNA was isolated from the indicated cell lines using a Qiagen RNeasy Mini Kit (Qiagen) according to the manufacturer’s instructions. RNA aliquots (1 µg) were then reverse transcribed using an iScript cDNA synthesis kit (Bio-Rad) according to standard protocols provided by the manufacturer. RT–qPCR was performed on cDNA samples using Brilliant III Ultra-Fast SYBR Green QPCR Master Mix (Agilent Technologies) and the signal was detected using an AriaMx real-time PCR system (Agilent Technologies). The fluorescence threshold value was calculated using Agilent Aria 1.6 software (Agilent Technologies). The PCR primers used were as follows: EHMT2 (forward, 5′-GAGAACATCTGCCTGCACTG-3′ and reverse, 5′-GTTGACAGCATGGAGGTCAC-3′); DDIT3 (forward, 5′-GGAAACAGAGTGGTCATTCCC-3′ and reverse, 5′-CTGCTTGAGCCGTTCATTCTC-3′); E-cadherin (forward, 5′-AAGTGACTGATGCTGATGCC-3′ and reverse, 5′-CACTGGATTTGTGGTGACGA-3′); N-cadherin (forward, 5′-AGAGGCTTCTGGTGAAATCG-3′ and reverse, 5′-TCCTTCATGCACATCCTTCG-3′); and ACTB (forward, 5′-ACTCTTCCAGCCTTCCTTCC-3′ and reverse, 5′-CAATGCCAGGGTACATGGTG-3′).

### FACS analysis

For the analysis using a Muse Annexin V and Dead Cell Assay kit (MCH100105, Merck), the cells were collected and incubated for 20 min at room temperature. For the analysis using a Muse Caspase 3/7 kit (MCH100108, Merck), the cells were collected and incubated with caspase-3/7 reagent (Merck) for 30 min in a humidified atmosphere with 5% CO_2_ at 37 °C. After the incubation, the cells were incubated with Caspase 7-AAD (Merck) for 5 min at room temperature. After the incubation, ~1 × 10^5^ cells were analyzed using a Muse cell analyzer (Merck) at TRCORE, Dong-eui University. The fluorescence-activated cell sorting (FACS) results were analyzed using Muse 1.6 analysis software (Merck).

### Migration and invasion assays

Transwell inserts were coated with a 2% gelatin solution and incubated at room temperature for 5 h for the migration assay. The gelatin-coated Transwell inserts (353097, BD Falcon) and invasion chambers (354480, Corning) were rehydrated in serum-free medium. Complete medium supplemented with 20% FBS (700 µl) served as a chemoattractant in the bottom chamber. A498 cells were seeded at 5 × 10^4^ cells/well, and Caki-1 cells were seeded at 1 × 10^5^ cells/well and incubated in the plates for 24 h at 37 °C with 5% CO_2_. At the end of the incubation period, the migrated and invaded cells were fixed with methanol for 5 min and stained with 0.1% CV for 5 min.

### Western blot analysis

The cells were washed once with phosphate-buffered saline (PBS) and then lysed in cold lysis buffer (50 mM Tris–HCl, pH 7.4, 150 mM NaCl, 1% Triton X-100, 0.1% SDS, 1 mM EDTA, 1 mM Na3VO4, 1 mM NaF and 1× protease inhibitor cocktail). The cell lysates were centrifuged at 14,000*g* for 20 min at 4 °C and then boiled in 5× sample buffer following the determination of the protein concentration (BSA, 23208, Thermo Fisher Scientific). The protein samples were subjected to western blot analysis using nitrocellulose membranes (1620145, Bio-Rad), blocking reagent (5% skim milk, 1 h, room temperature), precast gels (456-1095, Bio-Rad) and the indicated antibodies at a 1:1,000 dilution ratio. The membranes were incubated with primary antibodies. The primary antibodies used for western blotting were as follows: rabbit anti-EHMT2 (ab185050, ABclonal), anti-PARP (9542, Cell Signaling Technology), anti-H3K9me2 (ab1220, Abcam) and anti-ACTB (SC-47778, Santa Cruz). Membranes were incubated with the primary antibodies at 4 °C (overnight). The membranes were then incubated with secondary antibodies (rabbit; SC-2357, Santa Cruz; mouse; SC-516102, Santa Cruz) at room temperature for 1 h, and an ECL solution (170-5060, Bio-Rad) was used for visualization. A chemiluminescence imaging system (Mini HD9; UVitec) was used for imaging.

### Immunocytochemistry

Cultured cells were washed three times with ice-cold PBS and then fixed with 4% paraformaldehyde at room temperature for 10 min. The cells were subsequently washed three times with ice-cold PBS. Afterward, the cells were permeabilized with 0.1% Triton X-100 (Sigma-Aldrich) in PBS for 10 min and washed three times with PBS. The cells were blocked with 5% BSA in PBS for 30 min. Fixed cells were incubated with an anti-DDIT3 antibody (ab11419, Abcam) overnight at 4 °C and stained with Alexa Fluor-conjugated secondary antibodies (Life Technologies). Fluorescence images were obtained using a CELENA S Digital Imaging System (Logos Biosystems).

### IHC

For immunohistochemistry (IHC), paraffin-embedded sections of the kidney tumor tissue array (KD485a, TissueArray) were processed in a microwave (90 °C) with antigen-retrieval solution (pH 9) (S2367, Dako), treated with a peroxidase-blocking reagent and then treated with a protein-blocking reagent (K130, X0909, Dako). The tissue sections were incubated with a rabbit anti-EHMT2 antibody (ab185050, ABclonal) and anti-DDIT3 antibody (ab11419, Abcam), followed by an incubation with an HRP-conjugated secondary antibody (Dako). Immunoreactivity was visualized using a chromogenic substrate (Liquid DAB Chromogen, Dako). Finally, the tissue samples were stained with Mayer’s hematoxylin solution (Hematoxylin QS, Vector Laboratories) for 5 s to discriminate the nucleus from the cytoplasm. After the mice were killed, the tumors and organs were collected and fixed with 10% formalin for 24 h. Then the fixed tissues were sectioned and embedded in paraffin. Tissue Section (4 μm) were deparaffinized and then stained with hematoxylin and eosin (H&E) according to a standard protocol. Images of the whole cross section were captured using an EasyScan slide scanner (Motic). Images were analyzed using Motic ImagePlus software (Motic).

### ChIP

Chromatin immunoprecipitation (ChIP) assays were performed with a Simple ChIP Plus Sonication Chromatin IP kit (56383, CST) according to the manufacturer’s instructions. A498 cells and Caki-1 cells were transfected with siCont and siEHMT2 for 48 h or treated with DMSO or BIX for 48 h or treated with Fb7-311 *L. rhamnosus* treatment for 24 h, crosslinked with 1% formaldehyde (Sigma-Aldrich) for 10 min at room temperature and quenched with 1× glycine for 5 min at room temperature. Next, the cells were washed with cold 1× PBS (containing 1× protease inhibitor cocktail) and lysed in 1× cell lysis buffer (containing 1× protease inhibitor cocktail). After nuclear extraction, the chromatin mixture was sonicated using a Bioruptor Pico sonication device (B01060010, Diagenode) with 20 cycles of 30 s ON and 30 s OFF to obtain 200–1,000-bp chromatin fragments. Sheared chromatin (approximately 5–10 µg) was incubated with 2 μg of anti-H3K9me2 (ab8895, Abcam) ChIP-grade antibody and normal rabbit IgG (2729, CST) antibody at 4 °C (overnight). After the overnight incubation, the complexes were incubated with 30 μl of ChIP-grade Protein G magnetic beads for 2 h at 4 °C. The complexes were subsequently washed, incubated with ChIP elution buffer for 30 min at 65 °C and then incubated with proteinase K for 2 h at 65 °C. After DNA purification using spin columns, the samples were analyzed by quantitative PCR using DDIT3 primers. The primers used were as follows: DDIT3 promoter region (P1) forward, 5′-GTTCAAAAGGCCTATGTGCCC-3′ and reverse, 5′-TAGTCGGTCGTGAGCCTCTTT-3′; DDIT3 (P2) forward, 5′-AGGCTGATCTCGAACTCCT-3′ and reverse, 5′-GGTATCCATCCACTGGGATTGT-3′.

### RNA-seq and analysis

Using a TruSeq RNA Sample Preparation Kit V2, total RNA was purified and a library was constructed, and Illumina HiSeq 2500 instruments (Illumina) were used for sequencing, with a read length of 2 × 100 bases. FastQC v.0.11.4 was used to determine the quality of the paired-end reads. Cutadapt v.1.15 and Sickle v. 1.33 were used to filter low-quality reads and adapters. Cufflinks version 2.2.1 was used to calculate fragments per kilobase of transcripts per million mapped reads (FPKM) values. Cuffdiff was used to select differentially expressed genes (DEGs) (fold change >2). All Gene Ontology (GO) and Kyoto Encyclopedia of Genes and Genomes (KEGG) pathway enrichment analyses were performed with the Database for Annotation, Visualization and Integrated Discovery (DAVID) v6.8 and ClueGO v2.5.5 in Cytoscape v3.7.1.

### 3D spheroid culture

To perform spheroid culture of A498 cell lines, ultra-low-attachment microplates were used (7007, Corning). A498 cells were seeded into the plates at a concentration of 5 × 10^4^ cells/well and incubated for 24 h. Subsequently, cells were transfected with siEHMT2, co-transfected with siDDIT3 and siEHMT2, treated with BIX, or treated with Fb7-311, incubated for an additional 48 h, and then observed under a microscope (CKX53, Olympus Corporation).

### Extraction of metabolites

The protein concentration of each sample was quantified using the Thermo BCA Protein Assay kit. Metabolite extraction was performed based on an initial protein quantity of 200 μg per sample. Metabolite extraction was performed by adjusting the initial volume of each sample based on a protein amount of 200 μg. Four times the volume of ice-cold 100% methanol was added to the volume corresponding to 200 μg of protein, followed by vortexing for 1 min and incubation at −20 °C for 1 h. After centrifugation at 14,000*g* for 10 min, the supernatant was transferred to a new 1.5 ml tube and completely dried using a speed-vac centrifugal vacuum concentrator (Vision Scientific). Dried metabolite contents were reconstituted in 100 µl of 0.1% formic acid in water. Reconstituted samples were then passed through a 0.22-µm cellulose acetate spin filters (Agilent) and then subjected to liquid chromatography–tandem mass spectrometry (LC–MS/MS) analysis.

### LC–MS/MS analysis

LC–MS/MS analysis for metabolomics was performed UltiMate 3000 RSLC nano LC system (Thermo Scientific) coupled to a Q Exactive mass spectrometer equipped with a nano-ESI source (Thermo Scientific). Metabolite mixtures were loaded via an Acclaim PepMap 100 trap column (C18, 3 μm, 100 Å, 75 μm × 2 cm, Thermo Scientific), and subsequent metabolite separation was performed using an Acclaim PepMap RSLC analytical column (C18, 2 μm, 100 Å, 75 μm × 25 cm, Thermo Scientific). The mobile phase solvents consisted of (A) 0.1% formic acid in water and (B) 0.1% formic acid in 80% acetonitrile, and the flow rate was fixed at 400 nl/min. The gradient of mobile phase was as follows: 4% solvent B in 5 min, 4–28% solvent B in 27 min, 28–50% solvent B in 14 min, 50–96% solvent B in 0.1 min, holding at 96% of solvent B in 3.9 min, 96–4% solvent B in 0.1 min, 4% solvent B in 9.9 min. Mass parameters were set as follows: positive mode, spray voltage; 2.4 kV, negative mode, spray voltage; −2.4 kV, capillary temperature; and 320 °C. The properties of the full MS/dd-MS2 were set up as follows: full-MS scans, 100 to 1,000 *m*/*z* of scan range, 70,000 of resolution, 3× 106 of AGC target and maximum IT of 100 ms. For MS2 scans, the following parameters were used: 17,500 of resolution, 1× 105 of AGC target, maximum IT was 100 ms, ±2 *m*/*z* of isolation width and NCE for dd-MS2 of 27.

### Metabolome data analysis

Obtained RAW files were processed using Compound Discoverer 3.3 (Thermo Fisher Scientific). An untargeted metabolomics workflow was used to perform retention time alignment and compound identification. The identification of compounds was performed using mzCloud and ChemSpider. In the obtained dataset, identification was matched with levels 2 and 3 by filtering using annotation implemented with Metabolomics Standards Initiative of the Metabolomics Society^33^. Level 2 matched the exact mass (10 ppm) with a fragmentation score over 80 of the mzCould database and level 3 matched with the exact mass (5 ppm) in an MS1 database (Chemspider). Redundant metabolites were removed based on average area intensity. The background peaks were removed based on the blank file analyzed with solvent A. Based on the filtered and removed data, statistical analysis was performed using MetaboAnalyst 6.0 (http://www.metaboanalyst.ca)^34^.

### Animal experiments

A498 cells were implanted into the flanks of 7-week-old female BALB/c-nu mice (Nara Biotech) to establish a xenograft mouse model. BIX was intraperitoneally injected into the mice three times a week. The tumor size was measured three times a week, and the tumor volume was calculated from the length (L), width (W) and height (H). On day 23, all the mice were killed. The animal experiments were approved by the Committee on Animal Experimentation of the Korea Research Institute of Bioscience and Biotechnology.

### Statistical analysis

To classify patients into two groups, we performed receiver operating characteristic analysis based on the gene expression value of EHMT2 and calculated the best cutoff value, defined as the point with the highest combined sensitivity and specificity. The Kaplan–Meier method was used to calculate the time to recurrence or to metastasis, and differences between the times were assessed using log-rank tests.

## Results

### Overexpression of EHMT2 in RCC and induction of apoptosis by EHMT2 knockdown

**Fig. 1:**
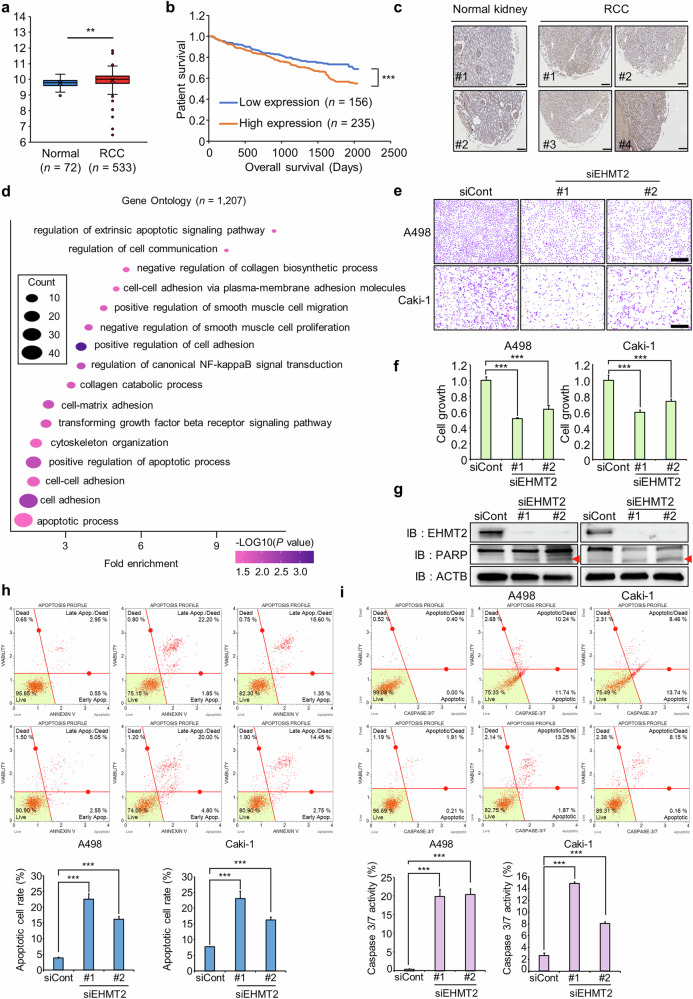
EHMT2 knockdown induces apoptosis in renal cancer cells. **a** EHMT2 expression in normal and RCC samples derived from TCGA database. *P* values were calculated using Student’s *t*-test (**P < 0.01). **b** Kaplan‒Meier plot showing that the overall survival rates of patients with low EHMT2 expression were substantially higher than those of patients with high EHMT2 expression in RCC tissues. *P* values were calculated using Student’s *t*-test (***P < 0.001). **c** Immunohistochemical staining for EHMT2. Kidney cancer tissues were purchased from TissueArray (https://www.tissuearray.com). Scale bar, 200 μm. **d** DAVID-based GO analysis of the RNA-seq results from the siEHMT2 (#1) and siCont groups, which included 1207 DEGs. **e**, **f** Cell growth assay after transfection with siEHMT2 and siCont for 48 h. **e** A498 and Caki-1 cells were fixed with 100% methanol and stained with the CV solution. Scale bar, 500 μm. **f** CCK-8 solution was added to the culture medium and the cells were incubated for 5 min at 37 °C. Cell growth was measured using a microplate reader (450 nm). The data are presented as the means ± s.d. of three independent experiments. *P* values were calculated using Student’s *t*-test (***P < 0.001). **g** Western blot analysis of cells transfected with siEHMT2 transfection using anti-EHMT2, anti-PARP and anti-ACTB antibodies. ACTB was used as the internal control in A498 and Caki-1 cells. **h** FACS analysis of Annexin V staining was performed after the cells were transfected with siEHMT2 or siCont. The lower right and upper right quadrants indicate early apoptotic cells and late apoptotic cells, respectively (top). Quantification of apoptosis: the data are presented as the means ± s.d. of three independent experiments. *P* values were calculated using Student’s *t*-test (***P < 0.001) (bottom). **i** FACS analysis using the Muse Caspase-3/7 working solution was performed after the cells were transfected with siEHMT2 or siCont. The upper right image shows the proportions of apoptotic and dead cells (top). Quantification of caspase-3/7 activity: the data are presented as the means ± s.d. of three independent experiments. *P* values were calculated using Student’s *t*-test (***P < 0.001) (bottom).

An analysis of RCC RNA-seq data derived from The Cancer Genome Atlas (TCGA) database revealed that EHMT2 expression was substantially upregulated in tumor tissues compared to normal tissues (Fig. 1a). IHC also confirmed that EHMT2 expression was higher in tumor tissues than in normal tissues (Fig. 1c). Furthermore, the prognostic analysis revealed that patients with high EHMT2 expression presented poor survival outcomes (Fig. 1b). Next, we designed EHMT2-specific siRNAs (siEHMT2 #1 and #2) to investigate the function of EHMT2 in RCC cells. Following siRNA treatment, RT‒qPCR and western blot analyses confirmed the reduction in EHMT2 expression (Supplementary Fig. 1a, b). Subsequently, the RNA-seq analysis of cells transfected with the EHMT2 siRNA or control siRNA (siCont) revealed 1,207 DEGs, including 865 upregulated and 342 downregulated genes. The GO analysis using DAVID indicated that EHMT2 knockdown was associated with terms related to ‘apoptosis process’, ‘cell migration’ and ‘cell‒cell adhesion’ (Fig. 1d). These findings suggest that EHMT2 overexpression is involved in the proliferation and metastasis of RCC.

CV staining and CCK-8 assays were performed following siEHMT2 transfection to determine the effect of EHMT2 knockdown on cell proliferation. As shown in Fig. 1e, f, the growth of the A498 and Caki-1 RCC cell lines was substantially suppressed by EHMT2 knockdown compared to siCont. In addition, western blot analysis further showed that EHMT2 knockdown increased the level of cleaved PARP (Fig. 1g). In addition, the FACS analysis revealed that, compared to siCont, EHMT2 knockdown increased the numbers of both early and late apoptotic cells, and caspase-3/7 activity was also increased (Fig. 1h, i). Collectively, these data indicated that EHMT2 knockdown induced apoptosis in RCC cells, corroborating the results of the GO analysis and supporting the involvement of EHMT2 in RCC cell growth.

### EHMT2 knockdown regulated the migration and invasion of A498 and Caki-1 cells

**Fig. 2:**
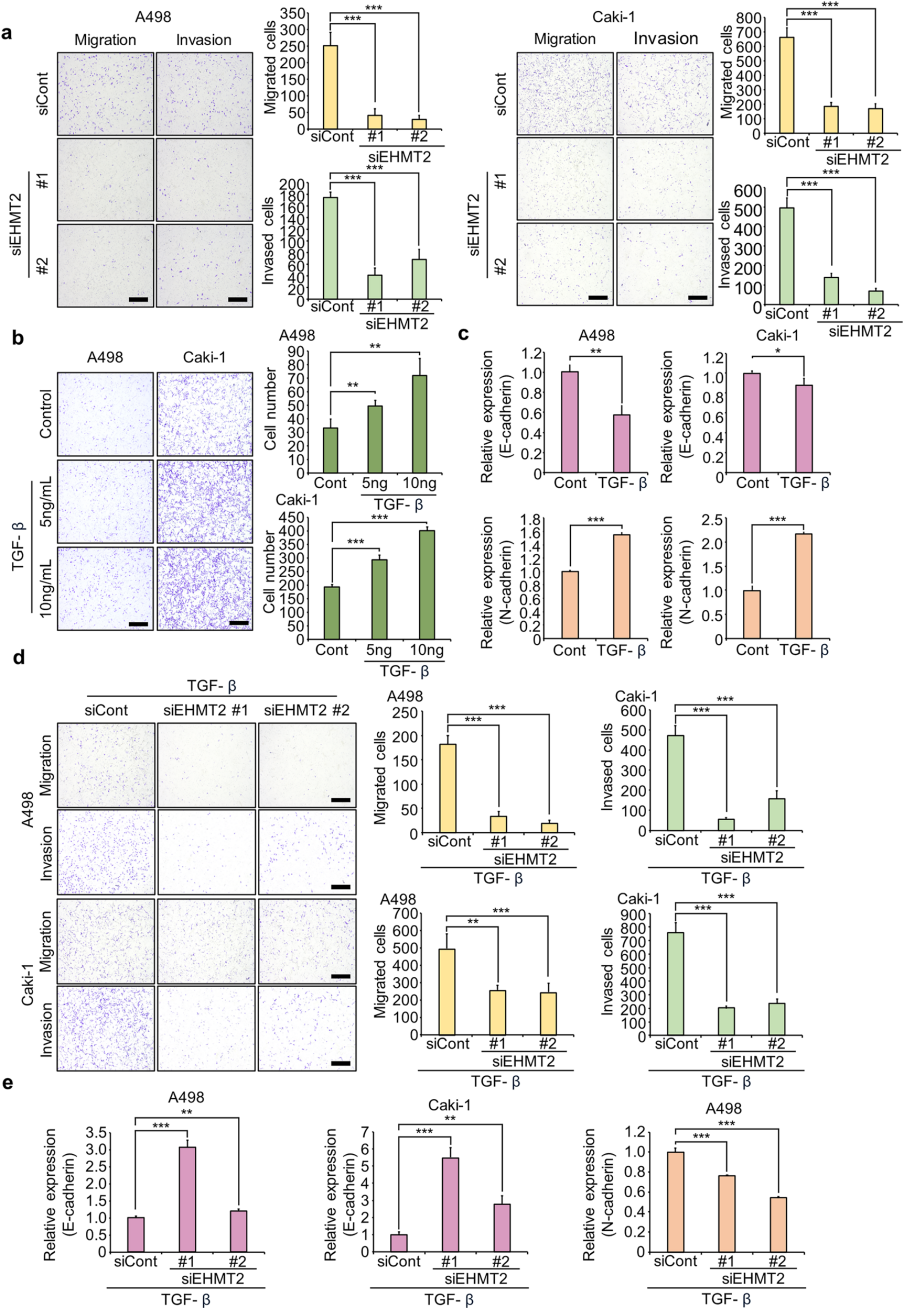
EHMT2 knockdown regulates the migration and invasion of renal cancer cell lines. **a** Migration and invasion assays were performed using the A498 and Caki-1 cell lines after EHMT2 knockdown. Cell migration and invasion assays were performed after 24 h (A498) and 48 h (Caki-1). The migrating/invading cells were stained with CV. Scale bar, 500 μm (left). Quantification of migrating/invading cells: the data are presented as the means ± s.d. of three independent experiments. *P* values were calculated using Student’s *t*-test (***P < 0.001) (right). **b** Migration assay of A498 and Caki-1 cells after treatment with TGF-β. The cell migration assay was performed after 24 h (A498) and 48 h (Caki-1). The migrating cells were stained with CV. Scale bar, 500 μm (left). Quantification of migrating cells: the data are presented as the means ± SDs of three independent experiments. *P* values were calculated using Student’s *t*-tests (**P < 0.01, ***P < 0.001) (right). **c** RT‒qPCR analysis of E-cadherin and N-cadherin expression in cells transfected with siEHMT2. The data are presented as the means ± s.d. of three independent experiments. *P* values were calculated using Student’s *t*-tests (*P < 0.05, **P < 0.01, ***P < 0.001). **d** Migration and invasion assays were performed using the A498 and Caki-1 cell lines after treatment with TGF-β and EHMT2 knockdown. Cell migration and invasion assays were performed after 24 h (A498) and 48 h (Caki-1). The migrating/invading cells were stained with CV. Scale bar, 500 μm (left). Quantification of migrating/invading cells: the data are presented as the means ± s.d. of three independent experiments. *P* values were calculated using Student’s *t-*tests (**P < 0.01, ***P < 0.001) (right). **e** RT‒qPCR analysis of E-cadherin and N-cadherin expression in cells transfected with siEHMT2. The data are presented as the means ± s.d. of three independent experiments. *P* values were calculated using Student’s *t*-tests (**P < 0.01, ***P < 0.001).

Cell migration and invasion assays were performed following EHMT2 knockdown to explore the involvement of EHMT2 in RCC metastasis. Compared to control cells, cells with EHMT2 knockdown exhibited substantially reduced migration and invasion abilities (Fig. 2a). Thus, these findings suggest that EHMT2 may regulate EMT to modulate the migration and invasion of RCC cell lines.

An in vitro EMT model was adopted by treating cells with transforming growth factor-beta (TGF-β) to further validate the role of EHMT2 in the EMT process^35^. TGF-β treatment increased the migration of RCC cells, accompanied by decreased expression of the epithelial marker E-cadherin and increased expression of the mesenchymal marker N-cadherin (Fig. 2b, c). When the EHMT2 siRNA was introduced into TGF-β-treated RCC cell lines, TGF-β-induced migration and invasion were suppressed by EHMT2 knockdown. This change was associated with increased expression of E-cadherin and decreased expression of N-cadherin (Fig. 2d, e). Thus, these findings suggest that EHMT2 is critically involved in the regulation of EMT in RCC cell lines and may represent a promising therapeutic target for preventing metastasis.

### BIX, a specific EHMT2 inhibitor, induced apoptosis and suppressed the migration/invasion of RCC cell lines

**Fig. 3:**
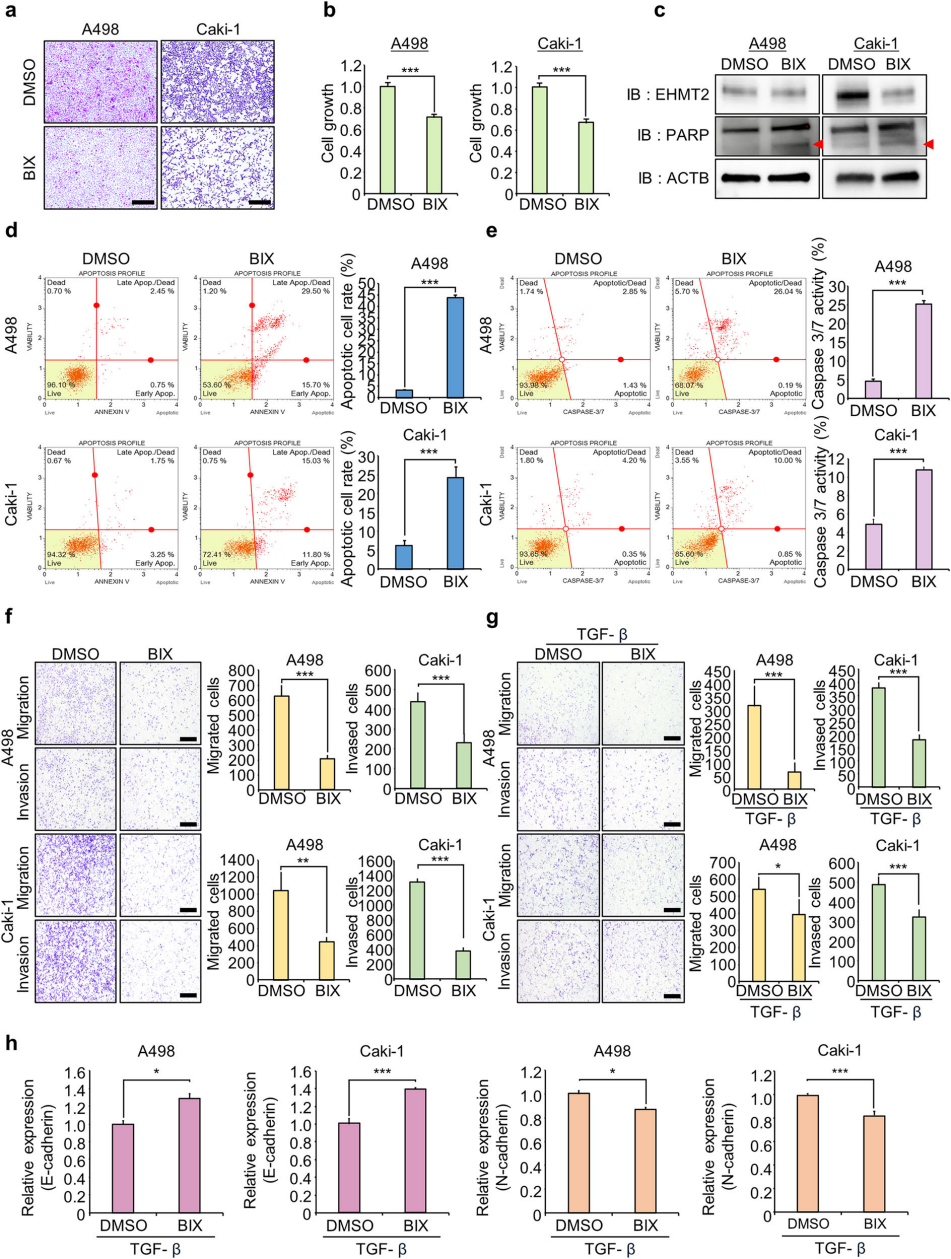
BIX, a specific EHMT2 inhibitor, regulates the migration and invasion of renal cancer cell lines. **a**, **b** Cell growth assay after treatment with BIX for 48 h: A498 and Caki-1 cells were fixed with 100% methanol and stained with a CV solution, scale bar, 500 μm (**a**); CCK-8 solution was added to the culture medium and the cells were incubated for 5 min at 37 °C. Cell growth was measured using a microplate reader (450 nm) (**b**). The data are presented as the means ± s.d. of three independent experiments. *P* values were calculated using Student’s *t*-test (***P < 0.001). **c** Western blot analysis of cells treated with BIX using anti-EHMT2, anti-PARP and anti-ACTB antibodies. ACTB was used as the internal control in A498 and Caki-1 cells. **d** FACS analysis of Annexin V staining was performed after BIX treatment. The lower right and upper right quadrants indicate early apoptotic cells and late apoptotic cells, respectively (left). Quantification of apoptosis: the data are presented as the means ± s.d. of three independent experiments. *P* values were calculated using Student’s *t*-test (***P < 0.001) (right). **e** FACS analysis using the Muse Caspase-3/7 working solution was performed after BIX treatment. The upper right image shows the proportions of apoptotic and dead cells (left). Quantification of caspase-3/7 activity: the data are presented as the means ± s.d. of three independent experiments. *P* values were calculated using Student’s *t*-test (***P < 0.001) (right). **f** Migration and invasion assays were performed in A498 and Caki-1 cells after BIX treatment. Cell migration and invasion assays were performed after 24 h (A498) and 48 h (Caki-1). The migrating/invading cells were stained with CV. Scale bar, 500 μm (left). Quantification of migrating/invading cells: the data are presented as the means ± s.d. of three independent experiments. *P* values were calculated using Student’s *t*-tests (**P < 0.01, ***P < 0.001) (right). **g** Migration and invasion assays were performed after the A498 and Caki-1 cell lines were treated with TGF-β and BIX. Cell migration and invasion assays were performed after 24 h (A498) and 48 h (Caki-1). The migrating/invading cells were stained with CV. Scale bar, 500 μm (left). Quantification of migrating/invading cells. The data are presented as the means ± s.d. of three independent experiments: *P* values were calculated using Student’s *t*-tests (*P < 0.05, ***P < 0.001) (right). **h** RT‒qPCR analysis of E-cadherin and N-cadherin expression in cells after BIX treatment. The data are presented as the means ± s.d. of three independent experiments. *P* values were calculated using Student’s *t*-tests (*P < 0.05, ***P < 0.001).

BIX, a specific inhibitor of EHMT2, has been widely used to study EHMT2 functions in various cancer types^36,37^. We assessed the effects of BIX on apoptosis and migration/invasion to investigate the therapeutic potential of EHMT2 inhibition in RCC. Proliferation assays indicated that BIX treatment substantially suppressed the growth of A498 and Caki-1 RCC cell lines, as shown by CV staining and CCK-8 assays (Fig. 3a, b). Western blot and FACS analyses confirmed the increase in cleaved PARP levels and apoptotic cell number after BIX treatment, along with increased caspase-3/7 activity (Fig. 3c–e). In addition, BIX treatment reduced cell migration and invasion (Fig. 3f). Moreover, TGF-β-induced migration and invasion were attenuated by BIX treatment, which also led to increased E-cadherin expression and decreased N-cadherin expression (Fig. 3g, h). These results suggest that EHMT2 is a promising therapeutic target for RCC and that the development of specific EHMT2 inhibitors may provide a novel strategy for treating RCC and preventing metastasis.

### DDIT3 was a direct target of EHMT2 in RCC

**Fig. 4:**
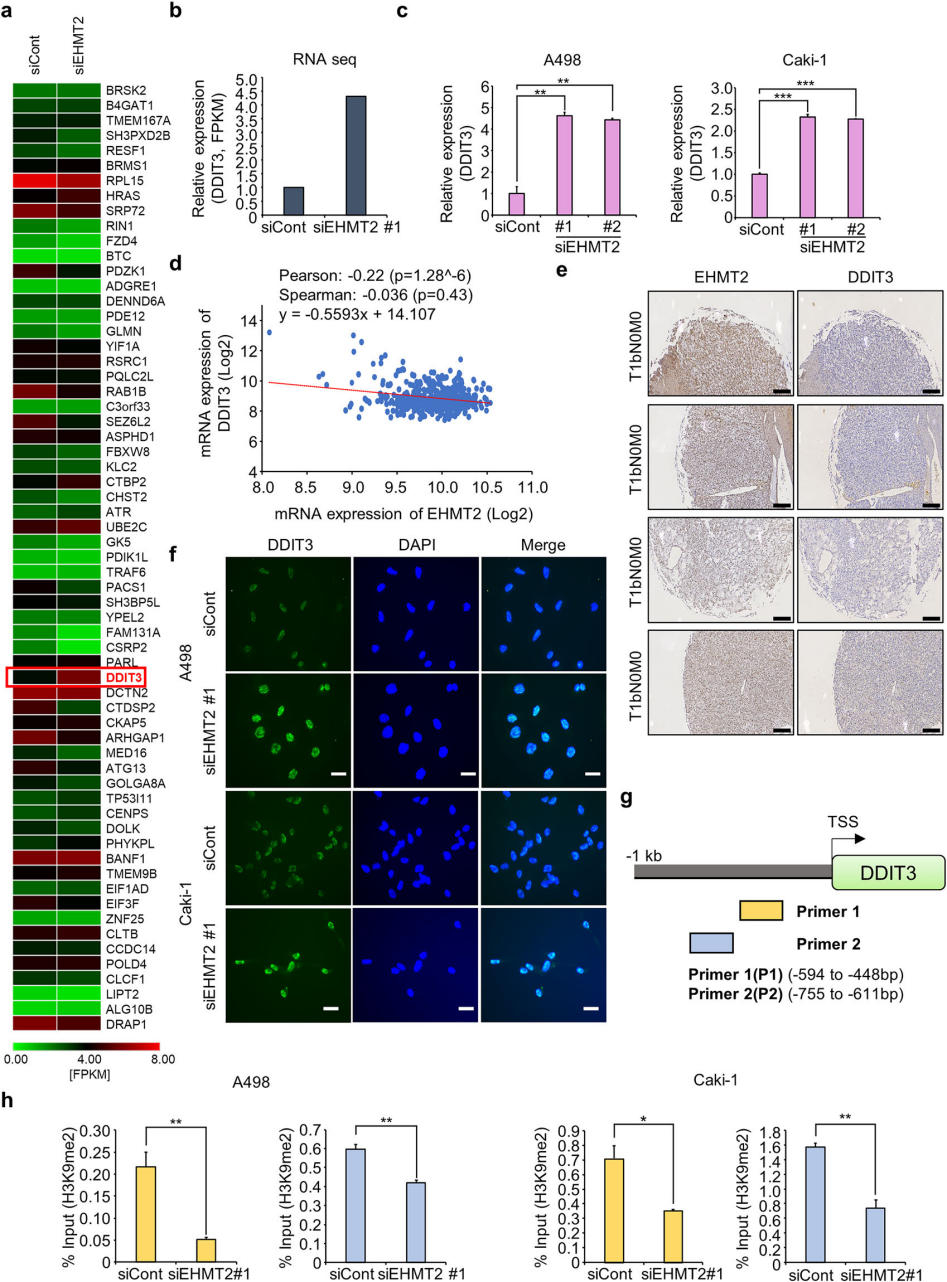
DDIT3 is a direct target of EHMT2 in renal cancer. **a** A heat map of RNA-seq data from siEHMT2- and siCont-transfected cells. **b** RNA-seq results for DDIT3 expression after EHMT2 knockdown. **c** RT‒qPCR analysis of DDIT3 expression in cells transfected with siEHMT2. The data are presented as the means ± s.d. of three independent experiments. *P* values were calculated using Student’s *t*-tests (**P < 0.01, ***P < 0.001). **d** Correlation analysis of the expression of the EHMT2 and DDIT3 genes derived from TCGA portal and using analysis of variance (ANOVA). **e** Immunohistochemical staining for EHMT2 and DDIT3. Kidney cancer tissues were purchased from TissueArray (https://www.tissuearray.com). Scale bar, 200 μm. **f** Immunocytochemical staining for DDIT3. A498 and Caki-1 cells transfected with siEHMT2 and siCont were fixed with 100% methanol and stained with an anti-DDIT3 antibody (Alexa Fluor 488, green) and DAPI (blue). Scale bar, 150 μm. **g** Graphical abstract of the ChIP primer design for the DDIT3 promoter region. **h** The ChIP assay was performed with an anti-H3K9me2 antibody. The result is shown as relative enrichment compared to the control in A498 and Caki-1 cells after siEHMT2 transfection. The data are presented as the means ± s.d. of three independent experiments. *P* values were calculated using Student’s *t*-tests (*P < 0.05, **P < 0.01).

**Fig. 5:**
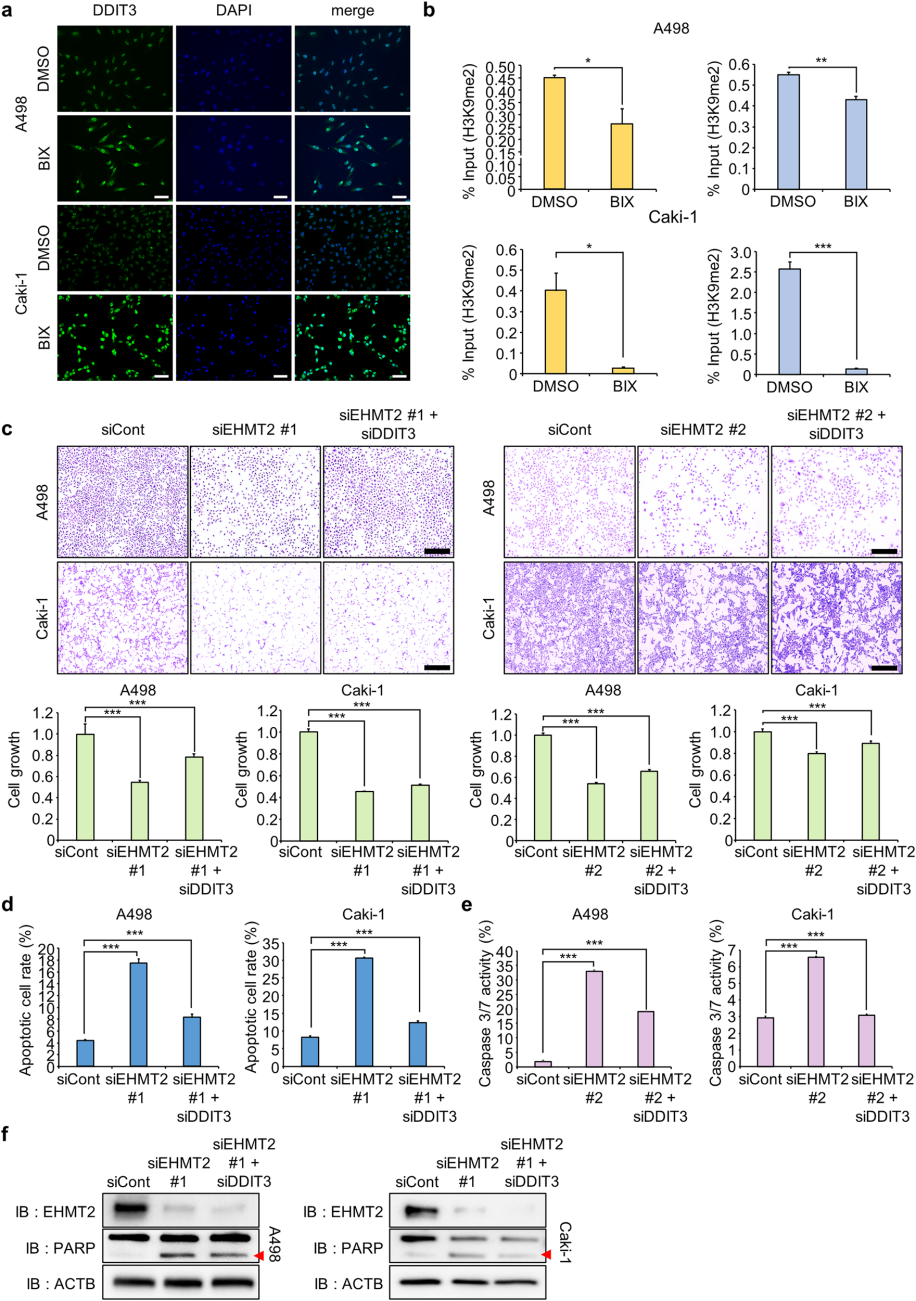
DDIT3 knockdown promotes apoptosis in A498 and Caki-1 cells. **a** Immunocytochemical staining for DDIT3. A498 and Caki-1 cells were treated with BIX, fixed with 100% methanol and stained with an anti-DDIT3 antibody (Alexa Fluor 488, green) and DAPI (blue). Scale bar, 300 μm. **b** The ChIP assay was performed with an anti-H3K9me2 antibody. The results are shown as relative enrichment compared to the control in A498 and Caki-1 cells after BIX treatment. The data are presented as the means ± s.d. of three independent experiments. *P* values were calculated using Student’s *t*-tests (*P < 0.05, **P < 0.01, ***P < 0.001). **c** Cell growth assay after cotransfection with siDDIT3 and siEHMT2 for 48 h. A498 and Caki-1 cells were fixed with 100% methanol and stained with a CV solution. Scale bar, 500 μm (top). CCK-8 solution was added to the culture medium and the cells were incubated for 5 min at 37 °C. Cell growth was measured using a microplate reader (450 nm). The data are presented as the means ± s.d. of three independent experiments. *P* values were calculated using Student’s *t*-test (***P < 0.001) (bottom). **d** FACS analysis of Annexin V staining was performed after cells were cotransfected with siDDIT3 and siEHMT2. Quantification of apoptosis: the data are presented as the means ± s.d. of three independent experiments. *P* values were calculated using Student’s *t*-test (***P < 0.001). **e** FACS analysis using the Muse Caspase-3/7 working solution was performed after cells were cotransfected with siDDIT3 and siEHMT2. Quantification of caspase-3/7 activity: the data are presented as the means ± s.d. of three independent experiments. *P* values were calculated using Student’s *t*-test (***P < 0.001). **f** Western blot analysis of cells cotransfected with siDDIT3 and siEHMT2 using anti-EHMT2, anti-PARP and anti-ACTB antibodies. ACTB was used as the internal control in A498 and Caki-1 cells.

We used a candidate approach to identify the direct targets of EHMT2 to elucidate how it regulates cell growth and metastasis in RCC (Supplementary Fig. 2a). Since EHMT2 is involved in the methylation of histone H3K9 to form heterochromatin^38,39^, genes that were upregulated after EHMT2 knockdown in the RNA-seq analysis were considered potential targets. Among them, DNA damage-inducible transcript 3 (DDIT3) was identified (Fig. 4a and Supplementary Fig. 2b). DDIT3, also known as C/EBP homologous protein (CHOP), is a key mediator of endoplasmic reticulum stress-induced apoptosis^40^. Under normal conditions, DDIT3 expression is maintained at low levels, but various cellular stresses, including endoplasmic reticulum stress, oxidative stress and nutrient fluctuations, can induce its expression^41,42^. Upon induction, DDIT3 promotes apoptosis through various mechanisms, including the upregulation of death receptor 5^43^. In cancer therapy, the activation of DDIT3 has been explored as a strategy to selectively induce cancer cell death^44,45^. Thus, we propose that the suppression of EHMT2 increases DDIT3 expression, thereby promoting apoptosis. RNA-seq and RT‒qPCR analyses confirmed increased DDIT3 expression following EHMT2 knockdown in A498 and Caki-1 cells compared to siCont-transfected cells (Fig. 4b, c). A correlation analysis of RCC RNA-seq data derived from TCGA portal revealed a negative correlation between EHMT2 and DDIT3 expression (Pearson’s correlation coefficient = −0.22) (Fig. 4d). IHC staining of tissue microarrays from RCC patients showed that tumors with high EHMT2 expression had lower DDIT3 expression, whereas those with low EHMT2 expression had higher DDIT3 expression (Fig. 4e). Immunocytochemistry also revealed increased DDIT3 expression following EHMT2 knockdown (Fig. 4f and Supplementary Fig. 2c). ChIP assays targeting the DDIT3 upstream region were performed using antibodies against dimethylated H3K9 to confirm the direct target of EHMT2 (Fig. 4g). Compared with that in the control cells, the H3K9 dimethylation status in A498 and Caki-1 cells was lower in the EHMT2 knockdown cells (Fig. 4h). BIX treatment similarly increased DDIT3 expression (Fig. 5a and Supplementary Fig. 3a) and decreased H3K9 dimethylation at the DDIT3 upstream region (Fig. 5b). Together, these results suggest that EHMT2 directly suppresses DDIT3 expression through the modulation of H3K9 methylation, contributing to apoptosis in RCC.

### The EHMT2–DDIT3 axis was a key regulator of apoptosis in RCC

A rescue experiment using a DDIT3-specific siRNA was conducted to confirm whether DDIT3 is a key regulator of apoptosis induced by EHMT2 knockdown. RT‒qPCR and imunocytochemistry confirmed the substantial knockdown of DDIT3 expression by siDDIT3 (Supplementary Fig. 3b, c). Cotransfection of siEHMT2 and siDDIT3 restored cell growth suppressed by siEHMT2 only, as evidenced by CV staining and the CCK-8 analysis (Fig. 5c). The FACS analysis further showed that siDDIT3 cotransfection reduced the apoptosis and caspase-3/7 activity induced by EHMT2 knockdown (Fig. 5d, e and Supplementary Fig. 3d, e). In addition, western blot analysis confirmed that the increase in cleaved PARP levels caused by EHMT2 knockdown was reduced upon siDDIT3 cotransfection (Fig. 5f). These results demonstrated that DDIT3 upregulation mediated the apoptosis induced by EHMT2 knockdown, establishing the EHMT2‒DDIT3 axis as a key regulator of RCC cell survival.

### *L. rhamnosus* Fb7-311 suppressed EHMT2 expression and induced apoptosis in RCC cell lines

**Fig. 6:**
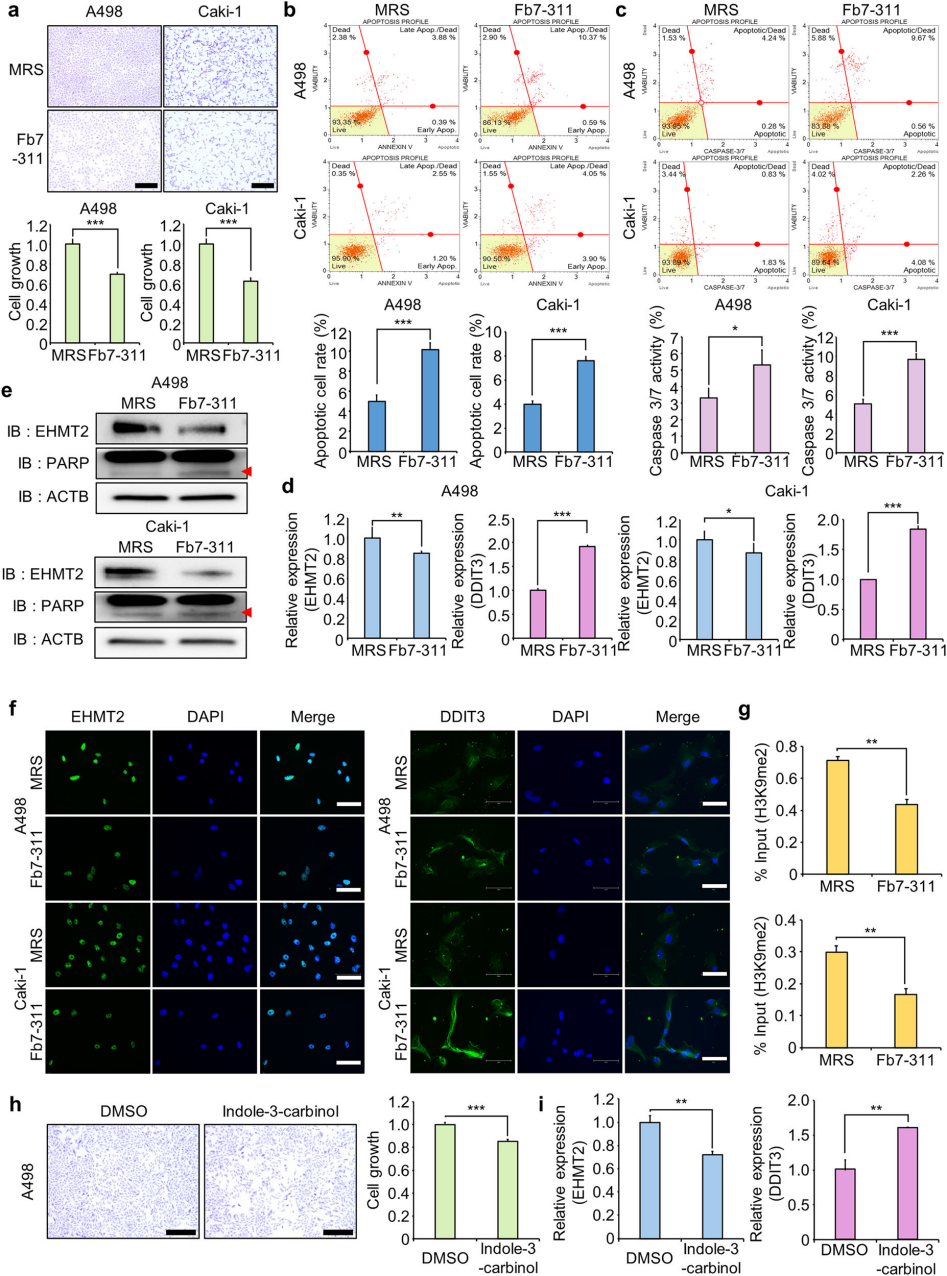
Fb7-311 regulates apoptosis in A498 and Caki-1 cells. **a** Cell growth assay after treatment with Fb7-311 for 24 h. A498 and Caki-1 cells were fixed with 100% methanol and stained with a CV solution. Scale bar, 500 μm (top). CCK-8 solution was added to the culture medium and the cells were incubated for 5 min at 37 °C. Cell growth was measured using a microplate reader (450 nm). The data are presented as the means ± s.d. of three independent experiments. *P* values were calculated using Student’s *t*-test (***P < 0.001) (bottom). **b** FACS analysis of Annexin V staining was performed after cells were treated with Fb7-311. The lower right and upper right quadrants indicate early apoptotic cells and late apoptotic cells, respectively (top). Quantification of apoptosis: the data are presented as the means ± s.d. of three independent experiments. *P* values were calculated using Student’s *t*-test (***P < 0.001) (bottom). **c** FACS analysis using the Muse Caspase-3/7 working solution was performed after cells were treated with Fb7-311. The upper right image shows the proportions of apoptotic and dead cells (top). Quantification of caspase-3/7 activity: the data are presented as the means ± s.d. of three independent experiments. *P* values were calculated using Student’s *t*-tests (*P < 0.05, ***P < 0.001) (bottom). **d** RT‒qPCR analysis of EHMT2 and DDIT3 expression after cells were treated with Fb7-311. The data are presented as the means ± s.d. of three independent experiments. *P* values were calculated using Student’s *t*-tests (*P < 0.05, **P < 0.01, ***P < 0.001). **e** Western blot analysis of cells treated with Fb7-311 using anti-EHMT2, anti-PARP and anti-ACTB antibodies. ACTB was used as the internal control in A498 and Caki-1 cells. **f** Immunocytochemical staining for EHMT2 and DDIT3. A498 and Caki-1 cells were treated with Fb7-311 fixed with 100% methanol and stained with an anti-DDIT3 antibody (Alexa Fluor 488, green) and DAPI (blue). Scale bar, 150 μm. **g** The ChIP assay was performed with an anti-H3K9me2 antibody on the DDIT3 promoter region. The result is shown as relative enrichment compared to the control in A498 and Caki-1 cells after Fb7-311 treatment. The data are presented as the means ± s.d. of three independent experiments. *P* values were calculated using Student’s *t*-test (**P < 0.01). **h** Cell growth assay after treatment with indole-3-carbinol for 72 h. A498 cells were fixed with 100% methanol and stained with a CV solution. Scale bar, 500 μm (left). CCK-8 solution was added to the culture medium and the cells were incubated for 5 min at 37 °C. Cell growth was measured using a microplate reader (450 nm). The data are presented as the means ± s.d. of three independent experiments. *P* values were calculated using Student’s *t*-test (***P < 0.001) (right). **i** RT‒qPCR analysis of EHMT2 and DDIT3 expression after cells were treated with indole-3-carbinol. The data are presented as the means ± s.d. of three independent experiments. *P* values were calculated using Student’s *t*-test (**P < 0.01).

Recent studies have reported that the gut microbiota can inhibit the growth and metastasis of various cancer types^46^. In addition, microbial metabolites such as propionate have been shown to destabilize the EHMT2 protein and increase apoptosis in colorectal cancer cells, highlighting the role of microbiota-derived metabolites in regulating cancer-related targets^10^. The screening of gut microbiota culture media revealed that the supernatant (Sup) of *L. rhamnosus* Fb7-311 suppressed RCC cell growth and modulated the EHMT2‒DDIT3 axis (Supplementary Fig. 4a, b). Treatment with Fb7-311 Sup inhibited the growth of A498 and Caki-1 cells, as shown by CV staining and CCK-8 assays (Fig. 6a). FACS analysis revealed increased apoptosis and caspase-3/7 activity following Fb7-311 Sup treatment (Fig. 6b, c). Fb7-311 Sup also reduced the migration and invasion of RCC cells, including TGF-β-induced migration and invasion, accompanied by increased epithelial marker expression and decreased mesenchymal marker expression (Supplementary Fig. 5a–c). RT‒qPCR and western blot analyses confirmed decreased EHMT2 expression and increased DDIT3 expression after Fb7-311 Sup treatment, along with increased cleaved PARP levels (Fig. 6d, e). Immunocytochemistry further verified the reduction in EHMT2 expression and increase in DDIT3 expression (Fig. 6f and Supplementary Fig. 5d). Moreover, to further validate the direct regulation of DDIT3 expression mediated by EHMT2 downregulation following Fb7-311 Sup treatment, we examined the H3K9me2 status at the DDIT3 promoter. Consistent with the effects observed following siEHMT2 knockdown and BIX treatment, exposure to the Fb7-311 Sup also resulted in a marked reduction of H3K9me2 levels at DDIT3. These results indicate that the decreased H3K9me2 status at the DDIT3 promoter is mediated by the reduction in EHMT2 expression induced by the Fb7-311 Sup (Fig. 6g).

Next, we performed metabolite profiling of the microbial culture supernatant to identify the specific metabolite(s) responsible for regulating the EHMT2–DDIT3 axis and suppressing the growth of renal cancer cell lines (Materials and methods). Based on the metabolite profiling results and a literature-based screening, we initially selected six candidate metabolites associated with cancer growth suppression: adenosine, sphingosine-1-phosphate, citric acid, cyclo (L-leu-L-pro), *N*-acetyl-L-histidine and DL-indole-3-lactic acid (Supplementary Fig. 6). To evaluate their growth-inhibitory effects on renal cancer cells, A498 cells were treated with each metabolite, followed by CCK and CV assays. As a result, growth-suppressive effects were observed (Fig. 6h and Supplementary Fig. 7).

As treatment with the Fb7-311 Sup induced changes in the EHMT2–DDIT3 axis, we further analyzed the effects of these six metabolites on EHMT2 downregulation and DDIT3 upregulation. Among them, only indole-3-carbinol substantially suppressed EHMT2 expression and enhanced DDIT3 expression (Fig. 6i). Collectively, our results indicate that indole-3-carbinol contained in the Fb7-311 culture supernatant induces EHMT2 downregulation, leading to subsequent DDIT3 upregulation and suppression of cell growth. Together, these results indicate that Fb7-311 Sup transcriptionally downregulates EHMT2, mimicking the effects of the EHMT2 siRNA, and suggest the potential application of microbiome-based therapies for RCC.

### EHMT2 as a therapeutic target for RCC

**Fig. 7:**
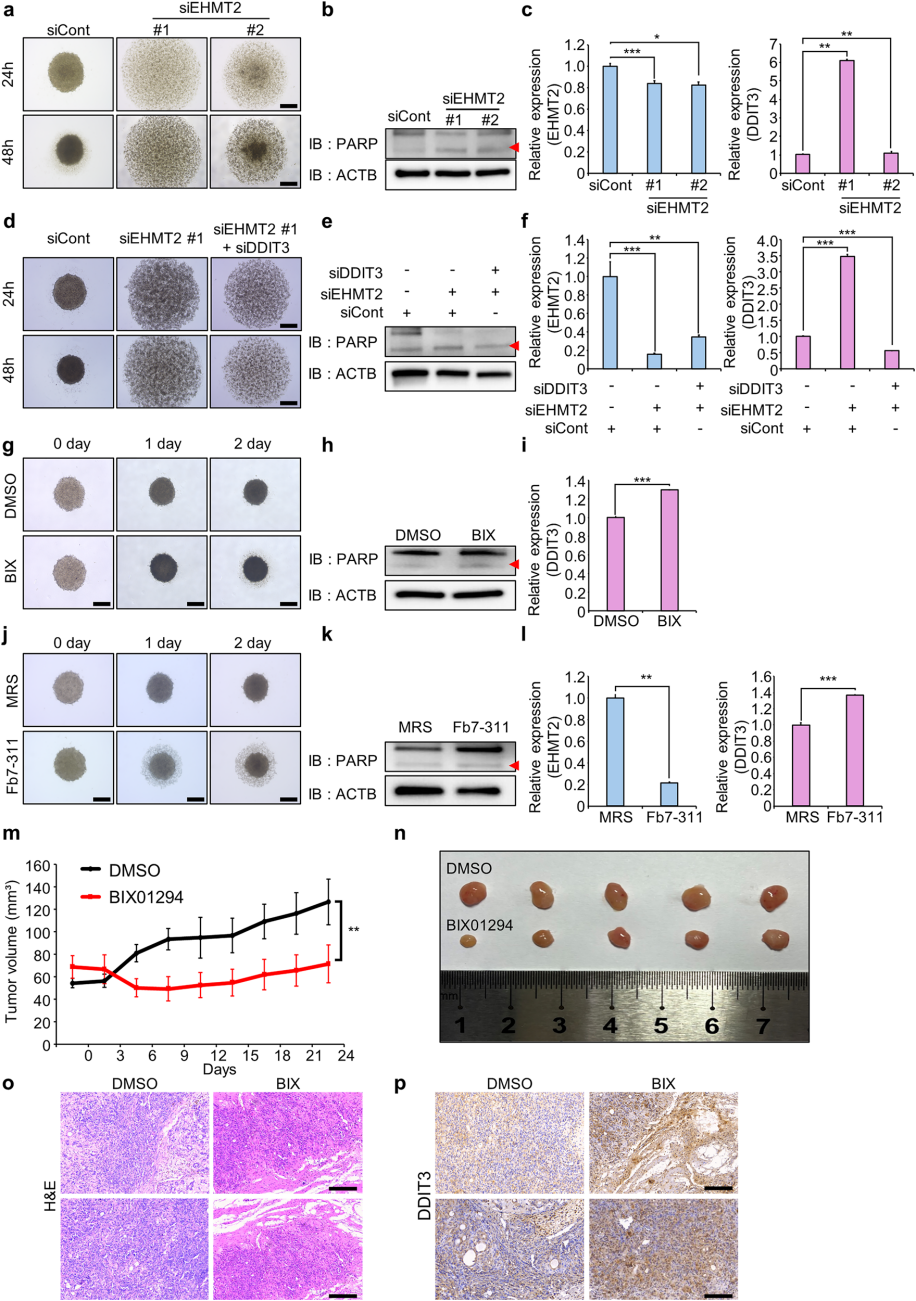
EHMT2 is a therapeutic target in RCC. **a** The 3D spheroid formation assay. The cells transfected with siEHMT2 and siCont were loaded onto ULA plates and incubated for 48 h. The cells were photographed under a microscope each day. Scale bar, 500 μm. **b** Western blot analysis of cells after EHMT2 knockdown using anti-PARP and anti-ACTB antibodies. ACTB was used as the internal control in A498 cell. **c** RT‒qPCR analysis of EHMT2 and DDIT3 expression after EHMT2 knockdown. The data are presented as the means ± s.d. of three independent experiments. *P* values were calculated using Student’s *t*-tests (*P < 0.05, **P < 0.01, ***P < 0.001). **d** The 3D spheroid formation assay. Cells cotransfected with siEHMT2 and siDDIT3 were loaded onto ULA plates and incubated for 48 h. The cells were photographed under a microscope each day. Scale bar, 500 μm. **e** Western blot analysis of cells cotransfected with siEHMT2 and siDDIT3 using anti-PARP and anti-ACTB antibodies. ACTB was used as the internal control in A498 cell. **f** RT‒qPCR analysis of EHMT2 and DDIT3 expression in cells cotransfected with siEHMT2 and siDDIT3. The data are presented as the means ± s.d. of three independent experiments. *P* values were calculated using Student’s *t*-tests (**P < 0.01, ***P < 0.001). **g** The 3D spheroid formation assay. After BIX was added to ULA plates, the cells were incubated for 48 h. The cells were then photographed under a microscope each day. Scale bar, 500 μm. **h** Western blot analysis of cells treated with BIX using anti-PARP and anti-ACTB antibodies. ACTB was used as the internal control in A498 cell. **i** RT‒qPCR analysis of DDIT3 expression in cells treated with BIX. The data are presented as the means ± s.d. of three independent experiments. *P* values were calculated using Student’s *t*-test (***P < 0.001). **j** The 3D spheroid formation assay. The cells were loaded onto ULA plates after treatment with Fb7-311 and incubated for 48 h. The cells were photographed under a microscope each day. Scale bar, 500 μm. **k** Western blot analysis of Fb7-311-treated cells using anti-PARP and anti-ACTB antibodies. ACTB was used as the internal control in A498 cell. **l** RT‒qPCR analysis of EHMT2 and DDIT3 expression in Fb7-311-treated cells. The data are presented as the means ± s.d. of three independent experiments. *P* values were calculated using Student’s *t*-tests (**P < 0.01, ***P < 0.001). **m**, **n** BIX treatment suppressed the growth of xenograft tumors in nude mice. Both the control and BIX were intraperitoneally injected three times a week after A498 cell implantation: tumor volumes (*P* values were calculated using two-way ANOVA (**P < 0.01)) **(m)** and macroscopic image of tumors on day 24 (**n**). **o** Representative H&E-stained mouse tumor sections. Scale bars, 200 μm. **p** Immunohistochemical staining for DDIT3 in mouse tumor sections. Scale bar, 200 μm.

Compared to traditional two-dimensional (2D) cultures, 3D spheroid models more closely mimic in vivo conditions and are frequently used to evaluate target function and therapeutic efficacy^47^. A498 cells formed well-defined spheroids after 48 h of culture on ultralow attachment (ULA) plates. By contrast, siEHMT2 transfection caused spheroid dissociation, indicating growth suppression similar to that observed in 2D models (Fig. 7a and Supplementary Fig. 8). Western blot and RT‒qPCR analyses confirmed increased cleaved PARP and DDIT3 levels in siEHMT2-transfected 3D spheroids (Fig. 7b, c). Cotransfection with siDDIT3 reversed the spheroid dissociation and reduced the increase in the level of cleaved PARP induced by siEHMT2 (Fig. 7d–f and Supplementary Fig. 8). BIX treatment also induced spheroid dissociation and increased cleaved PARP and DDIT3 levels (Fig. 7g–i and Supplementary Fig. 8). Similarly, Fb7-311 Sup treatment induced spheroid dissociation, increased cleaved PARP levels, and upregulated DDIT3 expression, consistent with the results from the 2D model (Fig. 7j–l and Supplementary Fig. 8). These findings suggest that Fb7-311 Sup, like siEHMT2 and BIX, effectively suppresses RCC growth through EHMT2 downregulation in 3D models, highlighting its potential as a microbiome-based therapeutic agent. In vivo xenograft assays further revealed a reduced tumor size following BIX treatment without substantial changes in mouse body weight (Fig. 7m, n and Supplementary Fig. 9). IHC analysis confirmed increased DDIT3 expression in BIX-treated tumors (Fig. 7o, p). Collectively, these results confirm that the modulation of the EHMT2‒DDIT3 axis suppresses RCC growth in both 3D spheroid and in vivo xenograft models, supporting the development of microbiome-based strategies targeting EHMT2 for RCC therapy.

## Discussion

A GO analysis of RNA-seq data obtained from cells with EHMT2 knockdown revealed the substantial involvement of EHMT2 in the regulation of both the proliferation and metastatic potential of RCC cell lines (Fig. 1d). EHMT2, a histone methyltransferase, contributes to heterochromatin formation by catalyzing the mono- and dimethylation of histone H3 at lysine 9 (H3K9me1/2)^38^. This chromatin modification is typically associated with transcriptional repression, suggesting that EHMT2 may silence genes involved in tumor suppression, including those that inhibit cancer cell proliferation or prevent metastatic dissemination. On the basis of this understanding, we initially aimed to identify downstream target genes of EHMT2 that simultaneously regulate tumor growth and metastasis. However, owing to the mechanistic overlap between growth suppression and metastasis inhibition, we strategically focused our analysis on apoptosis-related genes that are directly repressed by EHMT2. Among these, we identified DDIT3, a proapoptotic transcription factor, as a key target that mediates the antitumor effects observed upon EHMT2 inhibition. Despite the identification of DDIT3 as a representative downstream effector, we recognize that the full spectrum of EHMT2-regulated genes in RCC is probably broader. Future studies incorporating integrative omics approaches, such as ChIP-seq to map EHMT2 binding sites or assay for transposase-accessible chromatin using sequencing (ATAC-seq) to assess chromatin accessibility, will be essential to comprehensively map the transcriptional networks governed by EHMT2. These studies not only expand our understanding of the therapeutic relevance of EHMT2 but also provide insights into the potential side effects or off-target impacts of its inhibition in clinical settings.

Emerging evidence has linked the gut microbiome to a variety of diseases, including cancer, in which it plays a multifaceted role in modulating tumor growth, metastatic potential, immune system regulation, and responsiveness to chemotherapy^17,48^. In particular, certain bacterial strains or their metabolites have been shown to exert anticancer effects by suppressing oncogenic signaling pathways or by enhancing host immune responses. This growing body of literature highlights the therapeutic potential of microbiome-derived agents, which are often associated with low toxicity and may synergize with existing treatments to improve efficacy while minimizing adverse effects. Inspired by these insights, we screened a library of gut microbial strains isolated from pediatric fecal samples to identify candidates capable of modulating RCC proliferation. From this screen, we identified Fb7-311 as a promising bacterial strain that robustly inhibits both RCC cell proliferation and metastasis. Mechanistic studies revealed that treatment with the culture supernatant of Fb7-311 led to the downregulation of EHMT2 mRNA expression, resulting in the upregulation of DDIT3 expression. This effect mirrored that observed with direct EHMT2 knockdown via siRNA or pharmacological inhibition using selective EHMT2 inhibitors, suggesting that metabolites secreted by Fb7-311 may act as functional mimetics of EHMT2 inhibition (Supplementary Fig. 4). Interestingly, our previous work showed that the microbiota-derived short-chain fatty acid propionate could reduce EHMT2 protein stability, thus suppressing its function^10^. However, unlike propionate, Fb7-311 Sup treatment appears to downregulate EHMT2 expression at the transcriptional level, implying a distinct and possibly further upstream regulatory mechanism. This distinction underscores the need to identify and characterize the specific bioactive metabolites within the Fb7-311 Sup responsible for modulating EHMT2 expression. Our metabolomic analysis identified indole-3-carbinol as a key metabolite present in the Fb7-311 Sup. Notably, treatment with indole-3-carbinol recapitulated the transcriptional regulation of the EHMT2–DDIT3 axis observed following Fb7-311 Sup exposure, suggesting that this metabolite is a major functional mediator of the observed epigenetic effects. However, the precise molecular mechanism by which indole-3-carbinol suppresses EHMT2 expression remains to be fully elucidated. Future studies employing integrated multiomics approaches, including RNA-seq, ChIP-seq and ATAC-seq, will be essential to dissect the regulatory landscape governing EHMT2 modulation by indole-3-carbinol. Collectively, these findings highlight the therapeutic potential of microbial metabolite-based strategies for selectively targeting EHMT2, thereby offering a promising avenue for the development of epigenetic cancer therapies with enhanced specificity and reduced systemic toxicity.

Although our in vitro data clearly demonstrated that the Fb7-311 culture supernatant suppressed both the growth and metastasis of RCC cell lines, the in vitro experimental system has several limitations. Under physiological conditions, microbial metabolites must undergo intestinal absorption and hepatic metabolism before they can reach distant organs such as the kidney. Therefore, the direct application of the bacterial supernatant to cultured RCC cells in vitro may not accurately reflect in vivo pharmacokinetics. In addition, to the in vivo pharmacodynamic aspects, several critical factors should be considered to bridge the gap between the in vitro findings and their in vivo implementation. First, it is necessary to evaluate the colonization potential of Fb7-311 in the human gut. Although Fb7-311 was originally isolated from neonatal feces, its ability to successfully colonize the gut microbiota of cancer patients following oral administration has not yet been investigated. Successful colonization of Fb7-311 within the reshaped microbial ecosystem of patients with cancer is a prerequisite for exerting its biological effects and would provide crucial evidence for the functional role of the gut microbiome in RCC. Second, it is essential to determine whether the metabolites produced by Fb7-311 in vitro are similarly generated in vivo. Substantial differences between the gut environment and the in vitro culture supernatant—including oxygen availability, physicochemical conditions, and overall ecological complexity—may lead to markedly distinct metabolite profiles. Thus, alongside colonization assessment, in vivo validation of indole-3-carbinol, which regulates the EHMT2–DDIT3 axis, is also required. Through these evaluations, a more accurate in vivo assessment of the inhibitory effects of Fb7-311 on renal cancer will become possible. Accordingly, further in vivo studies using animal models are warranted to assess the bioavailability, pharmacokinetics and therapeutic efficacy of Fb7-311 and its metabolites under more physiologically relevant conditions, thereby addressing this limitation.

A fundamental component of any anticancer drug development pipeline is the thorough validation of therapeutic targets, which includes the elucidation of the mode of action and assessment of safety^49^. In this study, we proposed the EHMT2‒DDIT3 signaling axis as a novel and promising therapeutic target in RCC. Our in vitro experiments showed that Fb7-311 Sup treatment modulated this axis and substantially suppressed tumor growth and metastasis. We extended our experiments beyond 2D monolayer cultures and employed a 3D spheroid model, which more closely mimics the in vivo tumor microenvironment, to validate the translational relevance of our findings. Unlike 2D cultures, 3D systems recreate critical features of the tumor niche, including cell organization and oxygen gradients, thereby providing a more appropriate platform for evaluating therapeutic efficacy^50^. We utilized 3D spheroid models derived from RCC cell lines to assess the antitumor effects of EHMT2 knockdown and Fb7-311 Sup treatment. These experiments confirmed the blocking effects observed in 2D cultures and revealed that the EHMT2–DDIT3 pathway was strong in RCC and could be targeted by drugs. More recently, the use of patient-derived organoids (PDOs) has garnered increasing attention as an advanced preclinical model for cancer research. PDOs retain the histological and molecular features of primary tumors and allow for personalized therapeutic testing^51^. Future studies employing RCC PDOs will be critical for confirming the clinical relevance of EHMT2 and Fb7-311 and for evaluating the variability of treatment response across patient populations.

Although Fb7-311 is a naturally occurring bacterial strain originally isolated from neonatal fecal samples, its application as a therapeutic agent in humans necessitates a careful evaluation of its safety. While strains derived from the infant gut may be assumed to be generally nonpathogenic, their clinical use—whether as live biotherapeutics or in the form of culture-derived metabolites—must be rigorously tested to exclude any organ-specific or systemic toxicity. Recent advances in stem cell-derived organoid technologies have enabled assessments of both therapeutic efficacy and toxicity in vitro using human-relevant systems^52,53^. In particular, kidney organoids and intestinal organoids generated from human pluripotent stem cells serve as valuable platforms for assessing potential off-target effects. In this context, we propose the use of these organoid systems to evaluate the safety profile of Fb7-311 Sup, which will be an essential step toward clinical translation.

In summary, we showed that EHMT2 is overexpressed in RCC and that its suppression results in reduced tumor cell growth and metastatic capability, primarily through the induction of DDIT3 expression. Building on this mechanistic insight, we identified Fb7-311, a microbiota-derived strain, as a novel therapeutic candidate that exerts its antitumor effects by downregulating EHMT2 and increasing DDIT3 expression. These findings identify EHMT2 as a promising molecular target in RCC and suggest that Fb7-311 or its active metabolites could serve as effective therapeutic agents (Supplementary Fig. 10). Furthermore, the combination of Fb7-311 with selective EHMT2 inhibitors may produce synergistic effects, thereby enhancing therapeutic efficacy.

## Supplementary information

Supplementary Information

## References

[1] Sung, H. et al. Global cancer statistics 2020: GLOBOCAN estimates of incidence and mortality worldwide for 36 cancers in 185 countries. *CA Cancer J. Clin.* **71**, 209–249 (2021). 33538338 10.3322/caac.21660

[2] Hsieh, J. J. et al. Renal cell carcinoma. *Nat. Rev. Dis. Primers* **3**, 1–19 (2017). 10.1038/nrdp.2017.9 PMC5936048 28276433

[3] Heng, D. et al. Outcomes of patients with metastatic renal cell carcinoma that do not meet eligibility criteria for clinical trials. *Ann. Oncol.* **25**, 149–154 (2014). 24356626 10.1093/annonc/mdt492 PMC4155479

[4] Chevinsky, M. et al. Pathological stage T3a significantly increases disease recurrence across all tumor sizes in renal cell carcinoma. *J. Urol.* **194**, 310–315 (2015). 25676433 10.1016/j.juro.2015.02.013 PMC4509968

[5] Dawson, M. A. & Kouzarides, T. Cancer epigenetics: from mechanism to therapy. *Cell* **150**, 12–27 (2012). 22770212 10.1016/j.cell.2012.06.013

[6] Yang, J. et al. Epigenetic regulation in the tumor microenvironment: molecular mechanisms and therapeutic targets. *Signal Transduct. Target. Ther.* **8**, 210 (2023). 37217462 10.1038/s41392-023-01480-x PMC10203321

[7] Seligson, D. B. et al. Global levels of histone modifications predict prognosis in different cancers. *Am. J. Pathol.* **174**, 1619–1628 (2009). 19349354 10.2353/ajpath.2009.080874 PMC2671251

[8] Rogenhofer, S. et al. Decreased levels of histone H3K9me1 indicate poor prognosis in patients with renal cell carcinoma. *Anticancer Res.* **32**, 879–886 (2012). 22399607

[9] Artal-Martinez de Narvajas, A. et al. Epigenetic regulation of autophagy by the methyltransferase G9a. *Mol. Cell. Biol.* **33**, 3983–3993 (2013). 23918802 10.1128/MCB.00813-13 PMC3811684

[10] Ryu, T. Y. et al. Human gut-microbiome-derived propionate coordinates proteasomal degradation via HECTD2 upregulation to target EHMT2 in colorectal cancer. *ISME J.* **16**, 1205–1221 (2022). 34972816 10.1038/s41396-021-01119-1 PMC9038766

[11] Chen, Y. et al. The role of histone methylation in the development of digestive cancers: a potential direction for cancer management. *Signal Transduct. Target. Ther.* **5**, 143 (2020). 32747629 10.1038/s41392-020-00252-1 PMC7398912

[12] Li, R.-G. et al. Histone methyltransferase G9a promotes the development of renal cancer through epigenetic silencing of tumor suppressor gene SPINK5. *Oxid. Med. Cell. Longev.* **2021**, 6650781 (2021). 34336110 10.1155/2021/6650781 PMC8294961

[13] Casciello, F. et al. G9a drives hypoxia-mediated gene repression for breast cancer cell survival and tumorigenesis. *Proc. Natl Acad. Sci. USA* **114**, 7077–7082 (2017). 28630300 10.1073/pnas.1618706114 PMC5502591

[14] Dong, C. et al. G9a interacts with Snail and is critical for Snail-mediated E-cadherin repression in human breast cancer. *J. Clin. Invest.* **122**, 1469–1486 (2012). 22406531 10.1172/JCI57349 PMC3314447

[15] Lehnertz, B. et al. The methyltransferase G9a regulates HoxA9-dependent transcription in AML. *Genes Dev.* **28**, 317–327 (2014). 24532712 10.1101/gad.236794.113 PMC3937511

[16] Cryan, J. F. et al. The microbiota-gut-brain axis. *Physiol. Rev.* **99**, 1877–2013 (2019). 31460832 10.1152/physrev.00018.2018

[17] Zheng, D., Liwinski, T. & Elinav, E. Interaction between microbiota and immunity in health and disease. *Cell Res.* **30**, 492–506 (2020). 32433595 10.1038/s41422-020-0332-7 PMC7264227

[18] Turnbaugh, P. J. et al. An obesity-associated gut microbiome with increased capacity for energy harvest. *Nature* **444**, 1027–1031 (2006). 17183312 10.1038/nature05414

[19] Boursier, J. et al. The severity of nonalcoholic fatty liver disease is associated with gut dysbiosis and shift in the metabolic function of the gut microbiota. *Hepatology* **63**, 764–775 (2016). 26600078 10.1002/hep.28356 PMC4975935

[20] Ni, J., Wu, G. D., Albenberg, L. & Tomov, V. T. Gut microbiota and IBD: causation or correlation?. *Nat. Rev. Gastroenterol. Hepatol.* **14**, 573–584 (2017). 28743984 10.1038/nrgastro.2017.88 PMC5880536

[21] Garrett, W. S. Cancer and the microbiota. *Science* **348**, 80–86 (2015). 25838377 10.1126/science.aaa4972 PMC5535753

[22] Derosa, L. et al. Gut bacteria composition drives primary resistance to cancer immunotherapy in renal cell carcinoma patients. *Eur. Urol.* **78**, 195–206 (2020). 32376136 10.1016/j.eururo.2020.04.044

[23] Helmink, B. A., Khan, M. W., Hermann, A., Gopalakrishnan, V. & Wargo, J. A. The microbiome, cancer, and cancer therapy. *Nat. Med.* **25**, 377–388 (2019). 30842679 10.1038/s41591-019-0377-7

[24] Gamallat, Y. et al. *Lactobacillus rhamnosus* induced epithelial cell apoptosis, ameliorates inflammation and prevents colon cancer development in an animal model. *Biomed. Pharmacother.* **83**, 536–541 (2016). 27447122 10.1016/j.biopha.2016.07.001

[25] Sharaf, L. K., Sharma, M., Chandel, D. & Shukla, G. Prophylactic intervention of probiotics (*L. acidophilus*, *L. rhamnosus* GG) and celecoxib modulate Bax-mediated apoptosis in 1, 2-dimethylhydrazine-induced experimental colon carcinogenesis. *BMC Cancer* **18**, 1111 (2018). 30424722 10.1186/s12885-018-4999-9 PMC6234654

[26] Owens, J. A. et al. *Lactobacillus rhamnosus* GG orchestrates an antitumor immune response. *Cell. Mol. Gastroenterol. Hepatol.* **12**, 1311–1327 (2021). 34111601 10.1016/j.jcmgh.2021.06.001 PMC8463873

[27] Salemi, R. et al. *Lactobacillus rhamnosus* GG cell-free supernatant as a novel anti-cancer adjuvant. *J. Transl. Med.* **21**, 195 (2023). 36918929 10.1186/s12967-023-04036-3 PMC10015962

[28] Zhao, L.-Y. et al. Role of the gut microbiota in anticancer therapy: from molecular mechanisms to clinical applications. *Signal Transduct. Target. Ther.* **8**, 201 (2023). 37179402 10.1038/s41392-023-01406-7 PMC10183032

[29] Krautkramer, K. A. et al. Diet–microbiota interactions mediate global epigenetic programming in multiple host tissues. *Mol. Cell* **64**, 982–992 (2016). 27889451 10.1016/j.molcel.2016.10.025 PMC5227652

[30] Cen, X. et al. Pan-cancer analysis shapes the understanding of cancer biology and medicine. *Cancer Commun.* **45**, 728–746 (2025). 10.1002/cac2.70008 PMC12328095 40120098

[31] Paul, B. et al. Influences of diet and the gut microbiome on epigenetic modulation in cancer and other diseases. *Clin. Epigenetics* **7**, 112 (2015). 26478753 10.1186/s13148-015-0144-7 PMC4609101

[32] Zhang, H. et al. Beyond the gut: the intratumoral microbiome’s influence on tumorigenesis and treatment response. *Cancer Commun.* **44**, 1130–1167 (2024). 10.1002/cac2.12597 PMC11483591 39087354

[33] Sumner, L. W. et al. Proposed minimum reporting standards for chemical analysis: chemical analysis working group (CAWG) metabolomics standards initiative (MSI). *Metabolomics* **3**, 211–221 (2007). 24039616 10.1007/s11306-007-0082-2 PMC3772505

[34] Pang, Z. et al. MetaboAnalyst 6.0: towards a unified platform for metabolomics data processing, analysis and interpretation. *Nucleic Acids Res.* **52**, W398–W406 (2024). 38587201 10.1093/nar/gkae253 PMC11223798

[35] Nachiyappan, A., Gupta, N. & Taneja, R. EHMT1/EHMT2 in EMT, cancer stemness and drug resistance: emerging evidence and mechanisms. *FEBS J.* **289**, 1329–1351 (2022). 34954891 10.1111/febs.16334

[36] Kim, H., Choi, S. Y., Lim, J., Lindroth, A. M. & Park, Y. J. EHMT2 inhibition induces cell death in human non-small cell lung cancer by altering the cholesterol biosynthesis pathway. *Int. J. Mol. Sci.* **21**, 1002 (2020). 32028644 10.3390/ijms21031002 PMC7037906

[37] Xu, L. et al. EHMT2 inhibitor BIX-01294 induces endoplasmic reticulum stress mediated apoptosis and autophagy in diffuse large B-cell lymphoma cells. *J. Cancer* **12**, 1011 (2021). 33442400 10.7150/jca.48310 PMC7797660

[38] Tachibana, M., Matsumura, Y., Fukuda, M., Kimura, H. & Shinkai, Y. G9a/GLP complexes independently mediate H3K9 and DNA methylation to silence transcription. *EMBO J.* **27**, 2681–2690 (2008). 18818694 10.1038/emboj.2008.192 PMC2572175

[39] Shinkai, Y. & Tachibana, M. H3K9 methyltransferase G9a and the related molecule GLP. *Genes Dev.* **25**, 781–788 (2011). 21498567 10.1101/gad.2027411 PMC3078703

[40] Chen, X., Shi, C., He, M., Xiong, S. & Xia, X. Endoplasmic reticulum stress: molecular mechanism and therapeutic targets. *Signal Transduct. Target. Ther.* **8**, 352 (2023). 37709773 10.1038/s41392-023-01570-w PMC10502142

[41] Bento, C., Andersson, M. K. & Åman, P. DDIT3/CHOP and the sarcoma fusion oncoprotein FUS-DDIT3/TLS-CHOP bind cyclin-dependent kinase 2. *BMC Cell Biol.* **10**, 89 (2009). 20017906 10.1186/1471-2121-10-89 PMC2804592

[42] Song, B., Scheuner, D., Ron, D., Pennathur, S. & Kaufman, R. J. Chop deletion reduces oxidative stress, improves β cell function, and promotes cell survival in multiple mouse models of diabetes. *J. Clin. Invest.* **118**, 3378–3389 (2008). 18776938 10.1172/JCI34587 PMC2528909

[43] Yamaguchi, H. & Wang, H.-G. CHOP is involved in endoplasmic reticulum stress-induced apoptosis by enhancing DR5 expression in human carcinoma cells. *J. Biol. Chem.* **279**, 45495–45502 (2004). 15322075 10.1074/jbc.M406933200

[44] Peng, F. et al. Regulated cell death (RCD) in cancer: key pathways and targeted therapies. *Signal Transduct. Target. Ther.* **7**, 286 (2022). 35963853 10.1038/s41392-022-01110-y PMC9376115

[45] Amiri, M., Molavi, O., Sabetkam, S., Jafari, S. & Montazersaheb, S. Stimulators of immunogenic cell death for cancer therapy: focusing on natural compounds. *Cancer Cell Int.* **23**, 200 (2023). 37705051 10.1186/s12935-023-03058-7 PMC10500939

[46] Vivarelli, S. et al. Gut microbiota and cancer: from pathogenesis to therapy. *Cancers* **11**, 38 (2019). 30609850 10.3390/cancers11010038 PMC6356461

[47] Abbas, Z. N., Al-Saffar, A. Z., Jasim, S. M. & Sulaiman, G. M. Comparative analysis between 2D and 3D colorectal cancer culture models for insights into cellular morphological and transcriptomic variations. *Sci. Rep.* **13**, 18380 (2023). 37884554 10.1038/s41598-023-45144-w PMC10603139

[48] Geller, L. T. et al. Potential role of intratumor bacteria in mediating tumor resistance to the chemotherapeutic drug gemcitabine. *Science* **357**, 1156–1160 (2017). 28912244 10.1126/science.aah5043 PMC5727343

[49] Damia, G. & D’Incalci, M. Contemporary pre-clinical development of anticancer agents–what are the optimal preclinical models?. *Eur. J. Cancer* **45**, 2768–2781 (2009). 19762228 10.1016/j.ejca.2009.08.008

[50] Bhattacharya, S., Calar, K. & de la Puente, P. Mimicking tumor hypoxia and tumor-immune interactions employing three-dimensional in vitro models. *J. Exp. Clin. Cancer Res.* **39**, 75 (2020). 32357910 10.1186/s13046-020-01583-1 PMC7195738

[51] Pernik, M. N. et al. Patient-derived cancer organoids for precision oncology treatment. *J. Pers. Med.* **11**, 423 (2021). 34067714 10.3390/jpm11050423 PMC8156513

[52] Qu, S. et al. Patient-derived organoids in human cancer: a platform for fundamental research and precision medicine. *Mol. Biomed.* **5**, 6 (2024). 38342791 10.1186/s43556-023-00165-9 PMC10859360

[53] Xie, N. et al. hPSCs-derived brain organoids for disease modeling, toxicity testing and drug evaluation. *Exp. Neurol.* **385**, 115110 (2025). 39667657 10.1016/j.expneurol.2024.115110

