# Supplementary material for: Gut microbiota modulation of epigenetic target EHMT2: Lacticaseibacillus rhamnosus Fb7-311 regulated renal cell carcinoma apoptosis and metastasis
Source: Exp Mol Med. 2026 Mar 5;58(3):739–54. doi: 10.1038/s12276-026-01659-6 (PMC13049033; doi:10.1038/s12276-026-01659-6)
Supplement: Supplementary file 1 — Supplementary Information [file 12276_2026_1659_MOESM1_ESM.pdf]

Supplementary Figures

Gut microbiota modulation of epigenetic target EHMT2: *Lacticaseibacillus rhamnosus* Fb7-311 regulated renal cell carcinoma apoptosis and metastasis

Jeongmin Lee[1,2†], Jinkwon Lee[1†], In Hwan Tae[1], Yunsang Kang[1,2], Jinsan Kim[1,2], Tae Young Ryu[1], Haneol Yang[1,2], Su-gi Lee[1], Kunhyang Park[1], Doo-Sang Park[1, 2], Cho-Rok Jung[1, 2], Jung Hwa Lim[1, 2] , Moo-Seung Lee[1,2*], Dae-Soo Kim[1,2*], Mi-Young Son[1,2,3*], and Hyun-Soo Cho[1,2,3*]

[1]Korea Research Institute of Bioscience and Biotechnology, Daejeon, 34141, Republic of Korea; [2]Korea University of Science and Technology, Daejeon, 34316, Republic of Korea; [3]School of Medicine, Sungkyunkwan University, Suwon 16419, Republic of Korea.

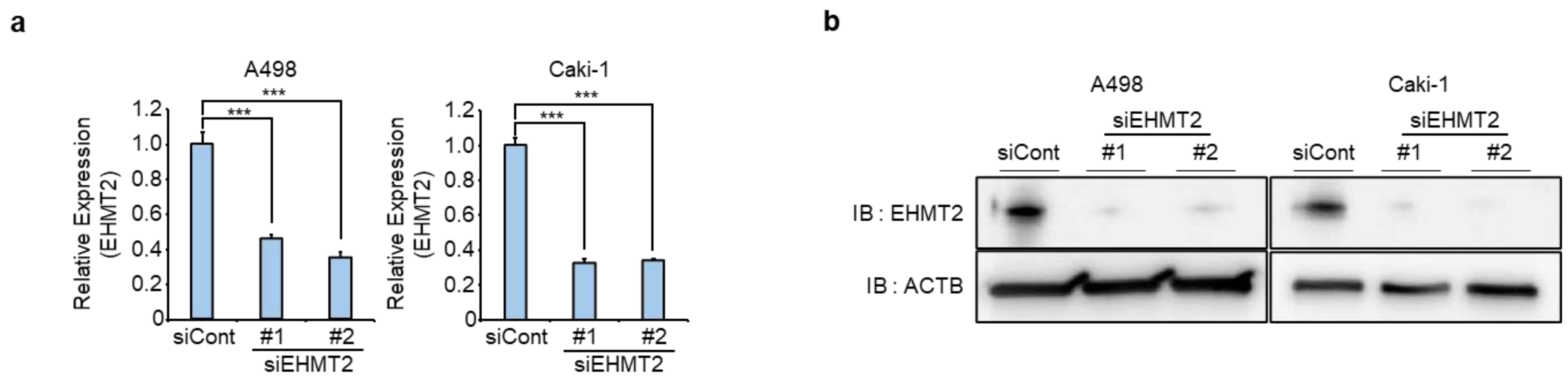

Supplementary Fig. 1 Reduction of EHMT2 expression by EHMT2 knockdown. a qRT–PCR analysis of EHMT2 expression in cells after EHMT2 knockdown. The data are presented as the means ± SDs of three independent experiments. *P* values were calculated using Student's *t* test (****P* < 0.001). **b** Western blot analysis of cells transfected with siEHMT2 transfection using anti-EHMT2 and anti-ACTB antibodies. ACTB was used as the internal control in A498 and Caki-1 cells.

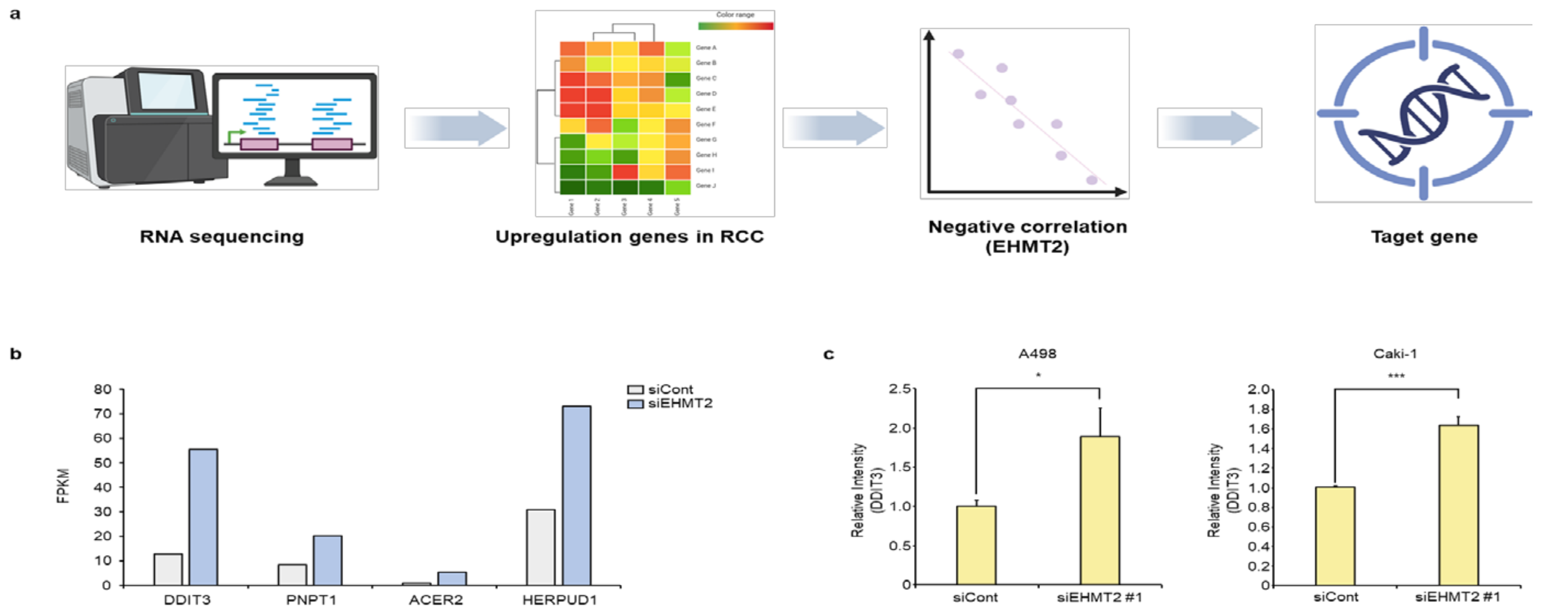

Supplementary Fig. 2 Finding DDIT3, a target of EHMT2. a Schematic summarizing the method used to identify the target of EHMT2 in kidney cancer. **b** RNA-seq results for DDIT3, PNPT1, ACER2 and HERPUD1 expression in cells after EHMT2 knockdown. **c** Quantification of DDIT3 expression via immunocytochemical analysis. The data are presented as the means ± SDs of three independent experiments. *P* values were calculated using Student's *t* tests (**P* < 0.05, ****P* < 0.001).

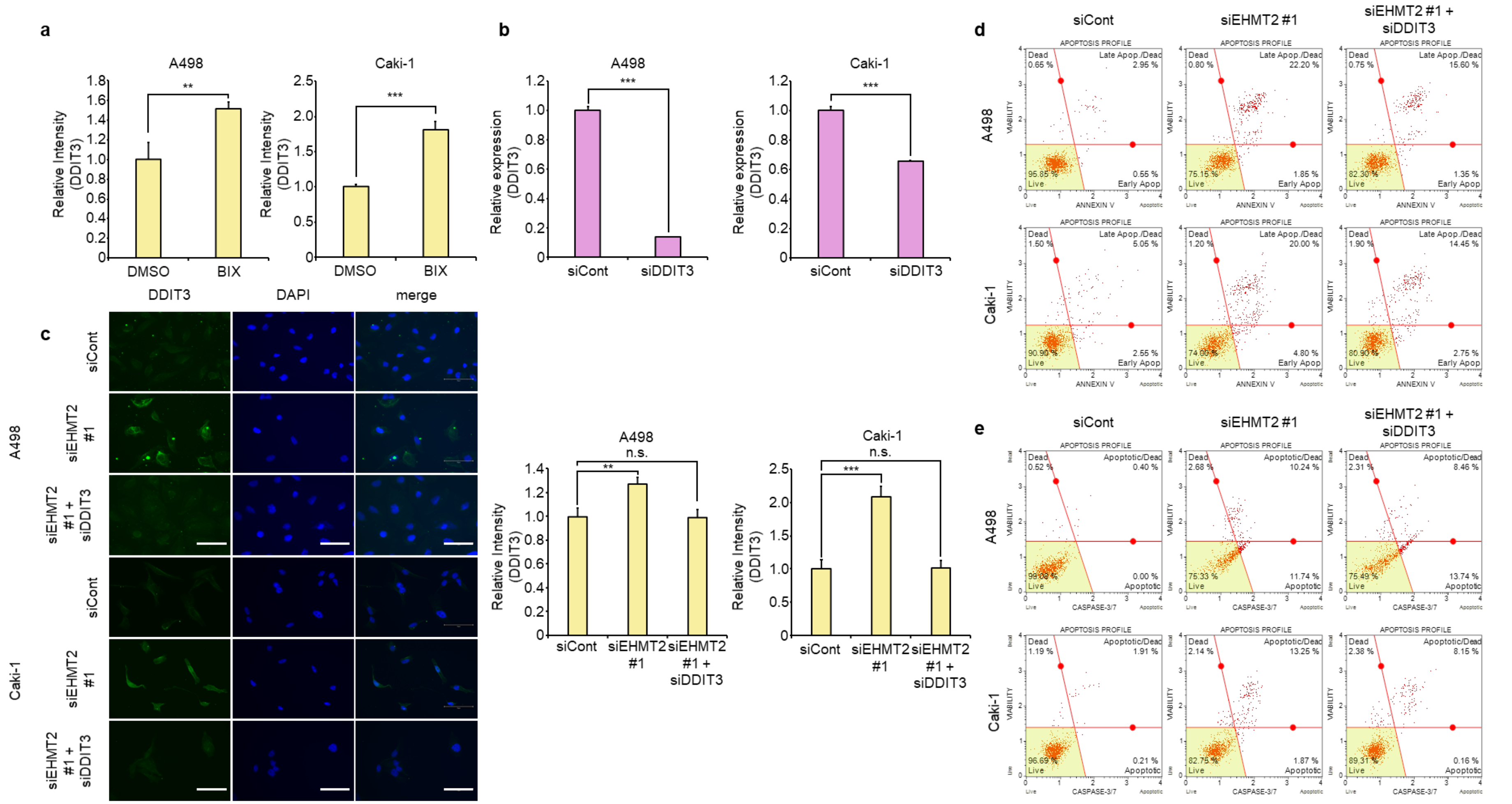

Supplementary Fig. 3 Change of DDIT3 expression level by DDIT3 knockdown. a Quantification of DDIT3 expression via immunocytochemical staining. The data are presented as the means ± SDs of three independent experiments. *P* values were calculated using Student's *t* tests (***P* < 0.01, ****P* < 0.001). **b** qRT–PCR analysis of DDIT3 expression after DDIT3 knockdown. The data are presented as the

means ± SDs of three independent experiments. *P* values were calculated using Student's *t* test (***$P < 0.001$). **c** Immunocytochemical staining for DDIT3. A498 and Caki-1 cells cotransfected with siEHMT2 and siDDIT3 were fixed with 100% methanol and stained with an anti-DDIT3 antibody (Alexa Fluor 488, green) and DAPI (blue). Scale bar, 150 μm (left panel). Quantification of DDIT3 expression via immunocytochemical staining. The data are presented as the means ± SDs of three independent experiments. *P* values were calculated using Student's *t* tests (n.s. not significant, **$P < 0.01$, ***$P < 0.001$) (right panel). **d** FACS analysis of Annexin V staining was performed after the cells were cotransfected with siEHMT2 and siDDIT3. The lower right and upper right quadrants indicate early apoptotic cells and late apoptotic cells, respectively. **e** FACS analysis using the Muse Caspase-3/7 working solution was performed after the cells were cotransfected with siEHMT2 and siDDIT3. The upper right panel shows the proportions of apoptotic and dead cells.

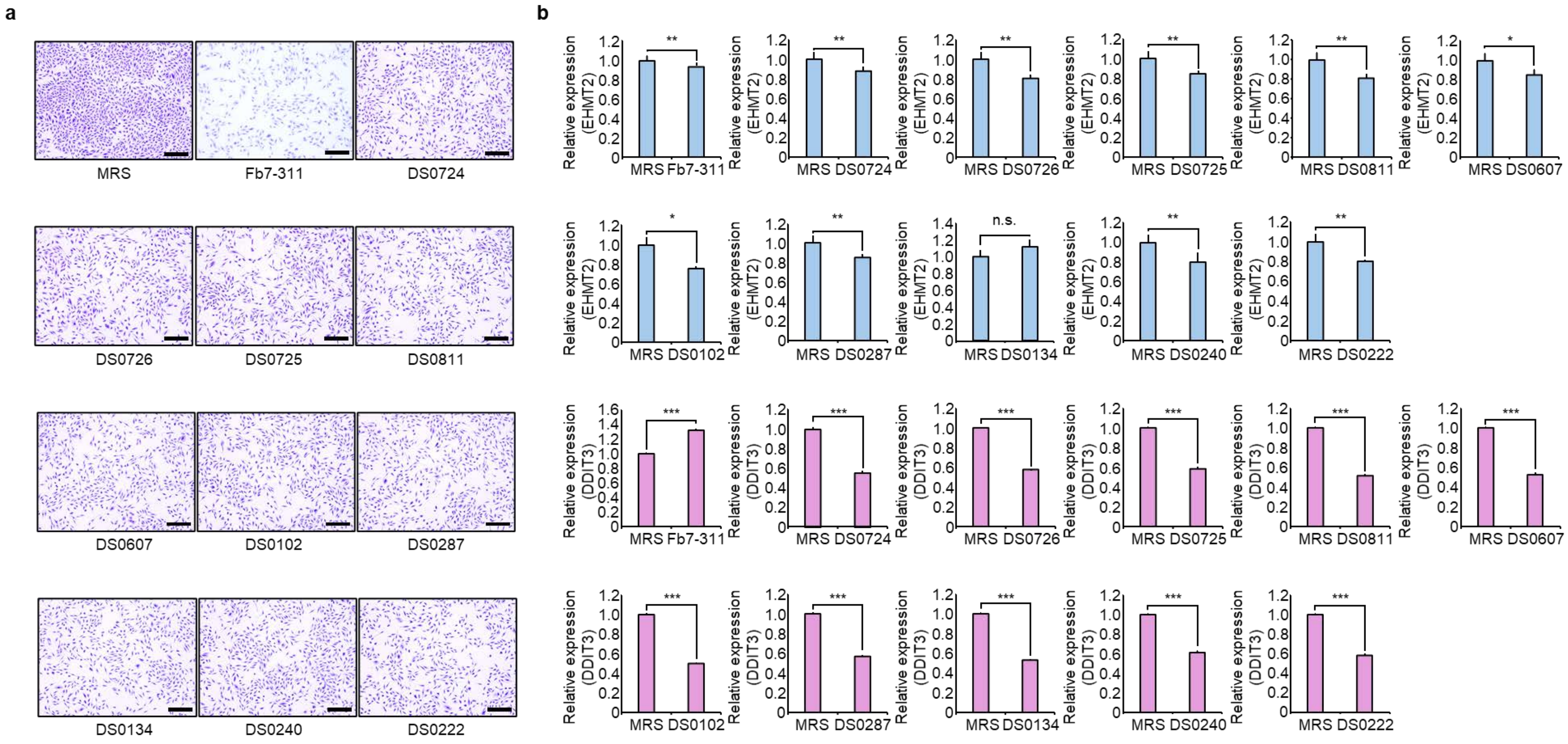

Supplementary Fig. 4 Screening of gut microbiota culture media. a Cell growth assay after treatment with the microbiota for 48 h. A498 cells were fixed with 100% methanol and stained with a CV solution. Scale bar, 500 μm. **b** qRT–PCR analysis of EHMT2 and DDIT3 expression after cells were treated with the microbiota. The data are presented as the means ± SDs of three independent experiments. *P* values were calculated using Student's *t* tests (n.s. not significant, *$P < 0.05$, **$P < 0.01$, ***$P < 0.001$).

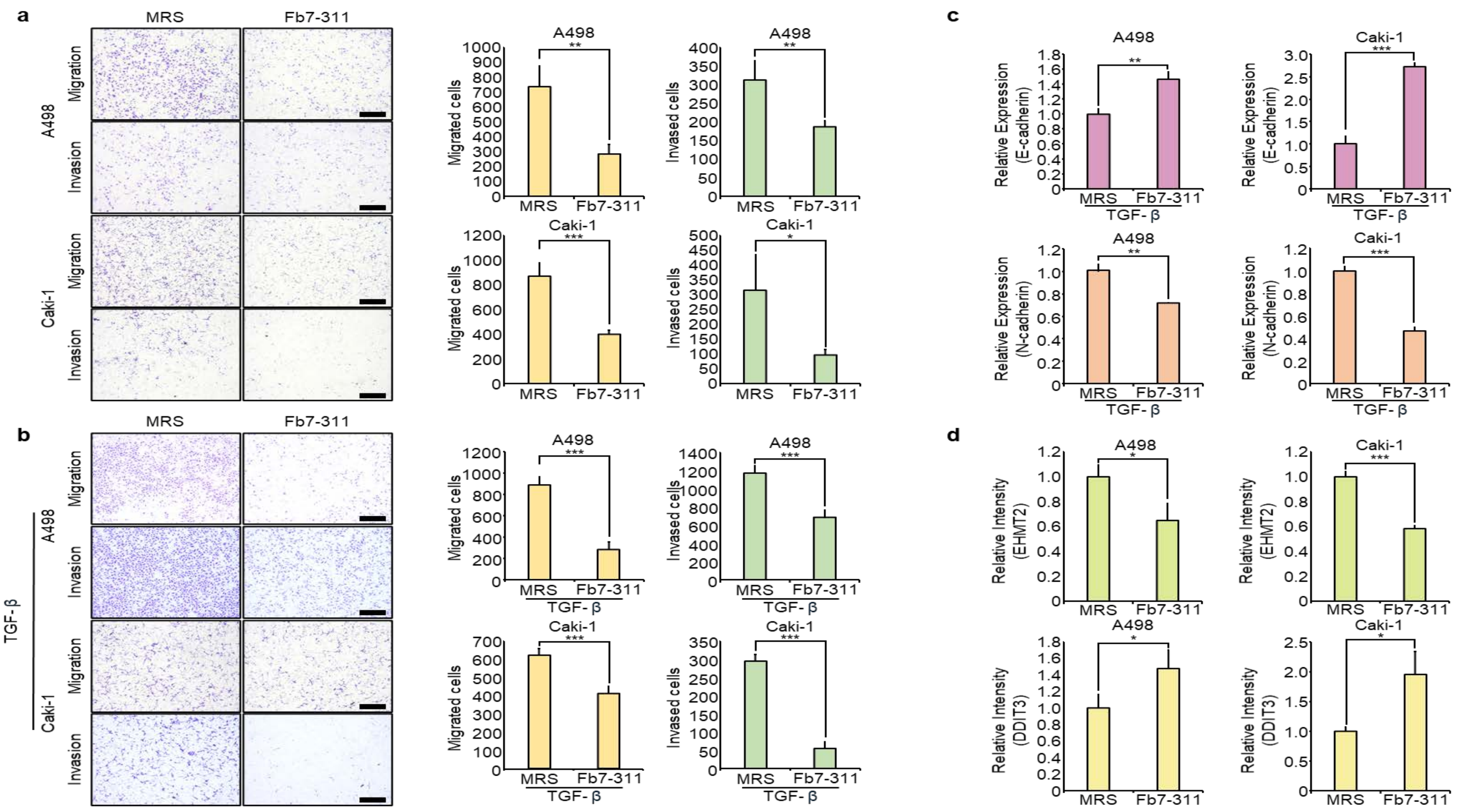

Supplementary Fig. 5 Fb7-311 Regulated cell migration and invasion in A498 and Caki-1 cell lines. a Migration and invasion assays of A498 and Caki-1 cells treated with Fb7-311 were performed. Cell migration and invasion assays were performed after 24 h (A498) and 48 h (Caki-1). The migrating/invading cells were stained with crystal violet. Scale bar, 500 μm (left panel). Quantification of migrating/invading

cells. The data are presented as the means ± SDs of three independent experiments. *P* values were calculated using Student's *t* tests (*$P < 0.05$, **$P < 0.01$, ***$P < 0.001$) (right panel). **b** Migration and invasion assays were performed in the A498 and Caki-1 cell lines after treatment with TGF-β and Fb7-311. Cell migration and invasion assays were performed after 24 h (A498) and 48 h (Caki-1). The migrating/invading cells were stained with crystal violet. Scale bar, 500 μm (left panel). Quantification of migrating/invading cells. The data are presented as the means ± SDs of three independent experiments. *P* values were calculated using Student's *t* test (***$P < 0.001$) (right panel). **c** qRT–PCR analysis of E-cadherin and N-cadherin expression after cells were treated with TGF-β and Fb7-311. The data are presented as the means ± SDs of three independent experiments. *P* values were calculated using Student's *t* tests (**$P < 0.01$, ***$P < 0.001$). **d** Quantification of EHMT2 and DDIT3 expression via immunocytochemical analysis. The data are presented as the means ± SDs of three independent experiments. *P* values were calculated using Student's *t* tests (*$P < 0.05$, ***$P < 0.001$).

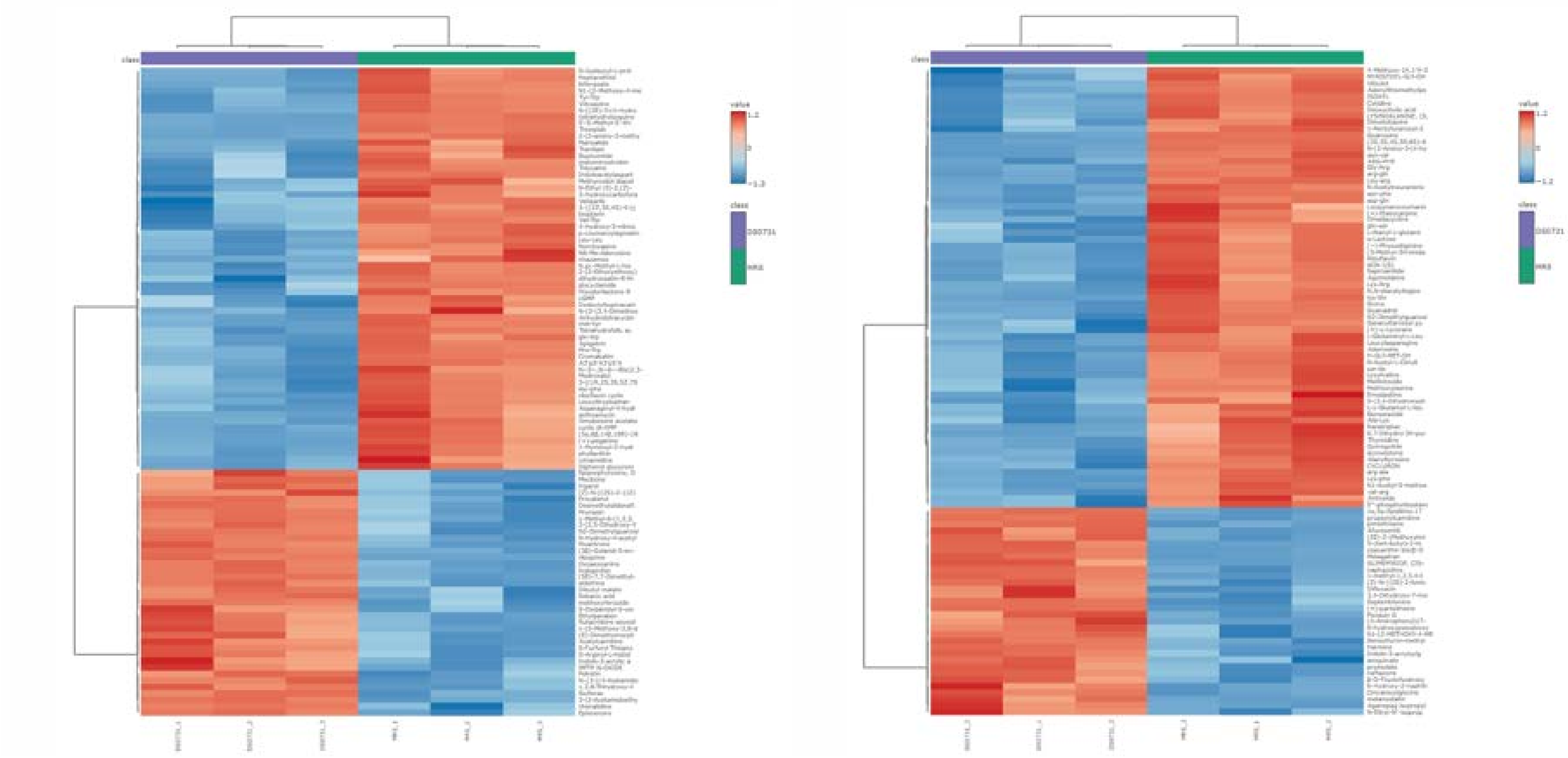

Supplementary Fig. 6 Heatmap of metabolites analysis. Left panel is positive mode and right panel is negative mode.

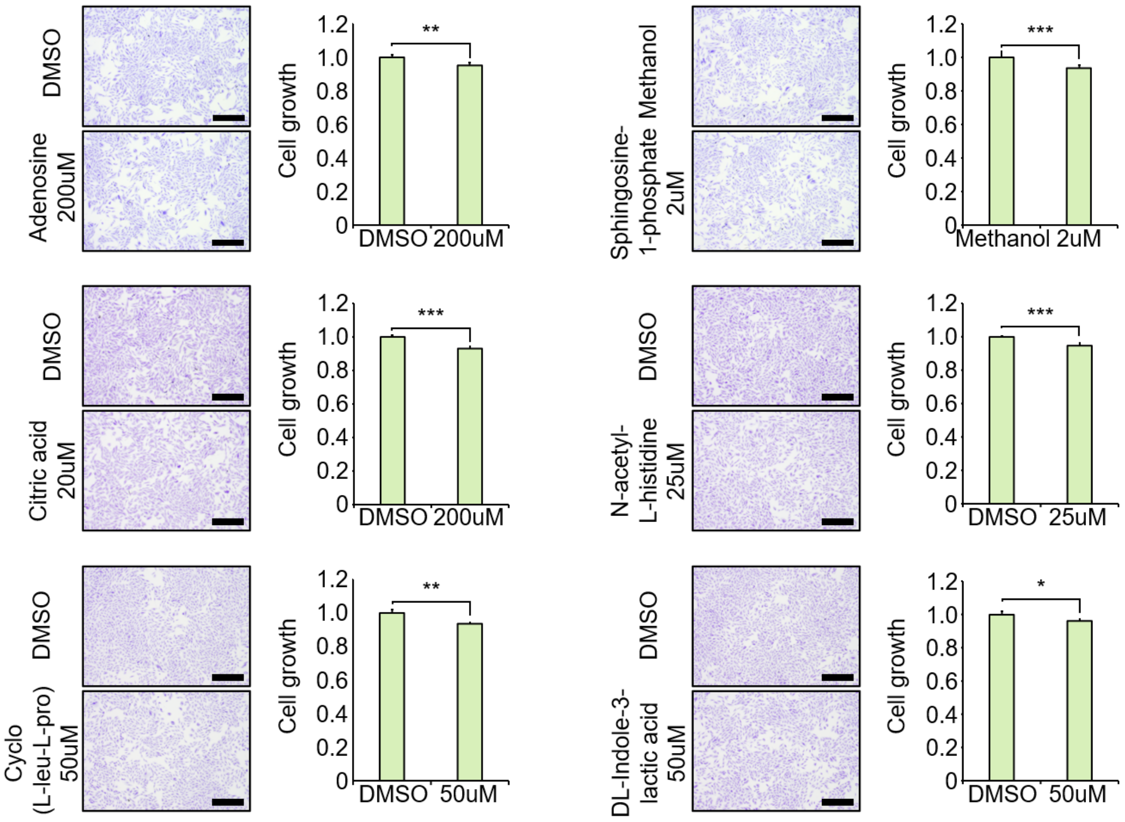

Supplementary Fig. 7 Cell growth assay after metabolites treatment. Cell growth assay after treatment Adenosine and Sphingosine-1-phosphate for 48h, Citric acid, Cyclo(L-leu-L-pro), N-acetyl-L-histidine and DL-Indole-3-lactic acid for 72h. A498 cells were fixed with 100% methanol and stained with the CV solution. Scale bar, 500 μm. CCK-8 solution was added to the culture medium, and the cells were incubated for 5 min at 37 °C. Cell growth was measured using a microplate reader (450 nm). The data are presented as the means ± SDs of three independent experiments. *P* values were calculated using Student's *t* tests (**P* < 0.05, ***P* < 0.01, ****P* < 0.001).

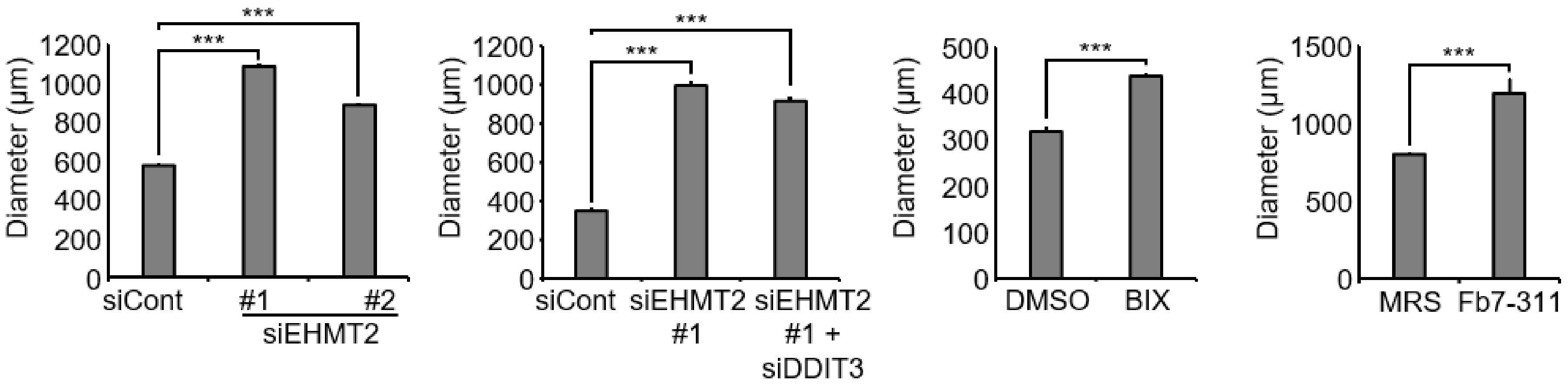

Supplementary Fig. 8 Quantification of spheroid diameter. Quantification of spheroid diameter of A498 cells after EHMT2 knockdown, cotransfected with siEHMT2 and siDDIT3, treated with BIX-01294 and Fb7-311. The data are presented as the means ± SDs of three independent experiments. *P* values were calculated using Student's *t* test (****P* < 0.001).

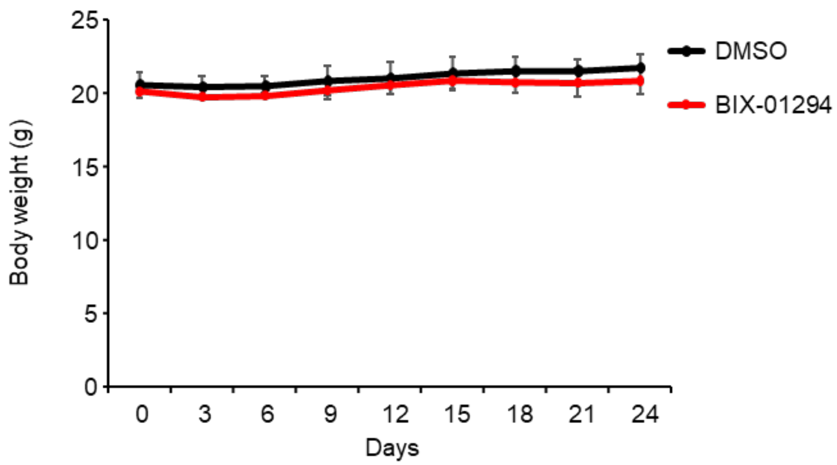

Supplementary Fig. 9 Body weight. Body weight measurements of tumor bearing mice during the treatment period.

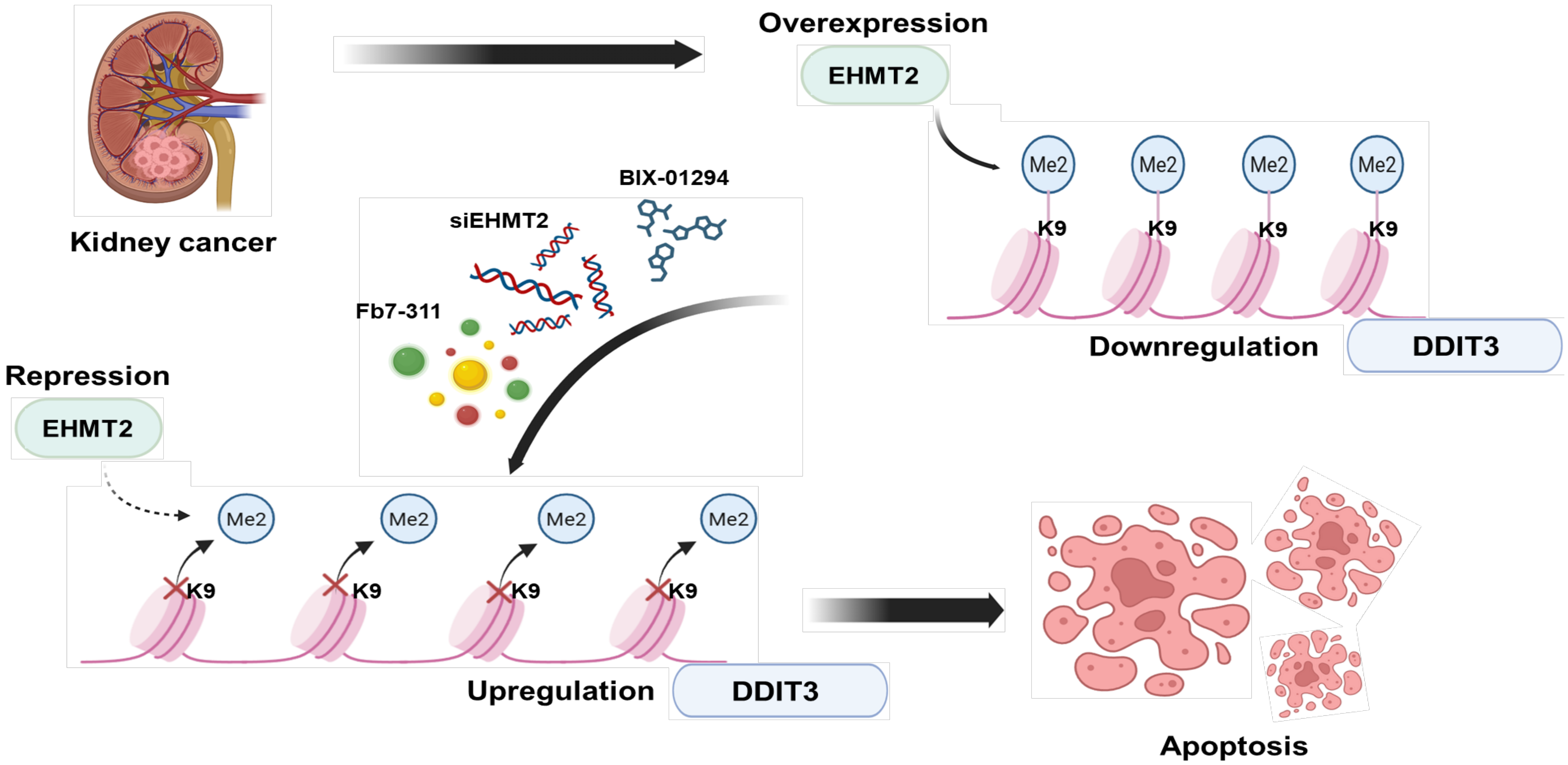

Supplementary Fig. 10 Schematic summarizing the role of EHMT2 in kidney cancer. EHMT2 overexpression suppressed DDIT3 expression by epigenetic regulation. EHMT2-specific inhibitors, siRNAs or microbiota could induce apoptosis and suppress migration and invasion via the upregulation of DDIT3 expression in kidney cancer.
